# Dynamics and microstructures in aqueous solutions of polyacrylamide and acrylamide monomers assessed by molecular dynamics simulations combined with optical Kerr effect spectroscopy

**DOI:** 10.1039/d6ra05577j

**Published:** 2026-08-17

**Authors:** Lin Wang, Huiqin Wang, Maolin Sha

**Affiliations:** a Department of Physics and Materials Engineering Hefei Normal University Hefei 230061 China mlsha@hfnu.edu.cn

## Abstract

Understanding the origin of the optical Kerr effect (OKE) signals in aqueous polymer systems is essential for interpreting the spectroscopic measurements of molecular dynamics in soft materials. Here, we employ molecular dynamics simulations combined with a dipole-induced dipole polarizability model to investigate aqueous acrylamide (AAm) and polyacrylamide (PAAm) solutions. Through the decomposition of the polarizability anisotropy time correlation function, we demonstrate that the OKE signals in monomer solutions arise primarily from solute reorientation, while in polymer solutions, they originate almost exclusively from water dynamics confined by the polymer network. The fast and intermediate relaxation components are nearly identical between the two systems, but the slowest component in the PAAm solutions is significantly delayed due to the much slower structural relaxation of the polymer chain, which modulates the water dynamics through hydrogen bonding. These results establish a quantitative framework for interpreting the OKE spectra of complex macromolecular systems.

## Abstract 2

The analysis of the OKE spectra of polymer solutions at the molecular level has long been challenging. This study combines experimental and simulated OKE spectra for comparative analysis, offering a new approach to the analysis of solution dynamics.

## Graphical abstract

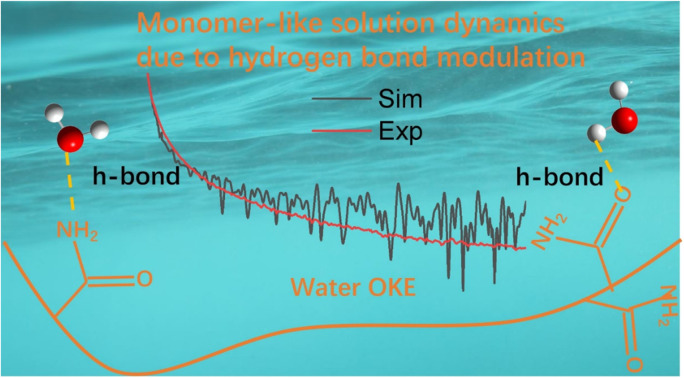

## 1. Introduction

Acrylamide (AAm) is a highly reactive organic compound capable of polymerization to form polyacrylamide, which is commonly used in a variety of industries. One of the applications of AAm is the fabrication of polyacrylamide hydrogels. Recent studies have demonstrated the potential of polyacrylamide hydrogels for applications in cell biology studies, gel electrophoresis, drug delivery systems and self-healing materials.^1–10^ In these hydrogel systems, the amide moiety of the monomer interacts strongly with water through hydrogen bonding. Previous experiments validated that the hydrogen bonding network of slowly confined water molecules appears as a single ensemble, without a difference between the core population and the interface water population at the polymer–water surface.^11^ Recent experiments have further demonstrated that the dynamic behavior of water molecules in such hydrogel systems is almost the same as that in monomer acrylamide–water mixtures.^12^ Hence, it is vital to observe the liquid dynamics of monomer acrylamide- and polyacrylamide-water mixtures in detail. Moreover, acrylamide contains a peptide linkage and can be regarded as a simple model to explore fundamental principles in biophysics as hydration effects and self-associated hydrogen bond (h bond) interactions are prevalent in biophysical processes.

Water is a simple, unique and important molecule in biophysics because of the extensive hydrogen bond networks and their rapid rearrangement.^13,14^ The dynamics of water molecules are largely impacted by solute structures in binary water solutions.^15,16^ In bulk water, the hydrogen bonding network completely determines the swift hopping dynamics and orientational dynamics. For acrylamide–water binary mixtures, the acrylamide molecule has hydrophilic –CO–NH_2_– groups, which interact strongly with water molecules to form hydrogen bonds. Besides, acrylamide is highly self-associated through directional hydrogen bonding with neighboring acrylamide molecules.^17^ All these strong hydrogen bonding interactions inevitably disrupt the hydrogen bonding network structures of bulk water. Several experiments and simulations have demonstrated that the slow dynamics of water in acrylamide–water mixtures depend on solution concentrations.^11,12^ However, the dynamical mechanism behind the water–water hydrogen bonding networks and acrylamide–water hydrogen bond interactions remains unclear. Hence, it is necessary to observe the detailed dynamics behaviors and molecular interactions in arcylamide- and polyacrylamide-water binary mixture systems. Although propionamide has a saturated backbone more similar to that of polyacrylamide, the key water-interaction site is the amide group, which is identical in both propionamide and acrylamide; consequently, their water interactions do not differ significantly. Furthermore, previous calculations^18^ reveal that their polarizabilities are highly comparable. Therefore, AAm is selected as the representative model due to its role as a direct precursor in polymerization.

Heterodyne-detected optical Kerr effect (OHD-OKE) spectroscopy is an ultrafast spectroscopic method that has been extensively applied to the study of liquid dynamics, including in bulk liquids, mixed solutions and confined liquids.^18–34^ OKE spectroscopy is a nonresonant pump–probe technique that measures the orientational dynamics of liquids. In a typical OKE experiment, an intense, linearly polarized pump pulse induces transient birefringence in the sample by minutely aligning the molecules or molecular subunits along the direction of the pump field. A weaker, time-delayed probe pulse, polarized at 45° relative to the pump, measures the decay of this birefringence, which reports on the system's return to an isotropic equilibrium distribution.^35–39^ Critically, the measured OKE signal, *R*(*t*), is directly proportional to the time derivative of the collective polarizability anisotropy time correlation function (PA-TCF), *ψ*(*t*).^38–40^ A key advantage of OKE spectroscopy is that the very same collective polarizability time correlation function can be rigorously calculated from molecular dynamics (MD) simulations.^40–44^ Thus, the convergence of OKE experiments and MD simulations—where the latter quantitatively reproduces the former and provides a microscopic dissection of the measured signal—offers a uniquely powerful and validated approach to elucidate the intricate relationships between molecular structure, intermolecular interactions, and emergent dynamical properties in systems ranging from simple polar liquids to complex hydrogels and concentrated electrolytes.^21,22,40^ In the present study, we leverage this consistency to investigate the dynamics of acrylamide and polyacrylamide solutions, where OKE experiments directly report on the species dynamics and MD simulations are employed to compute the polarizability correlation function and other more comprehensive structural information and dynamic properties, allowing for a direct, parameter-free comparison and detailed molecular-level interpretation.

## 2. Theoretical background and methods

### 2.1 OKE theoretical background

In a binary mixture consisting of two distinct molecular species, u (acrylamide) and v (water), the collective polarizability tensor Π_sum_ is given by the sum of the molecular and interaction-induced contributions from both components as follows:

1 Π_sum_ = Π^M^ + Π^I^ = (Π^M^_u_ + Π^M^_v_) + (Π^I^_u_ + Π^I^_v_)

The interaction-induced part is calculated using the dipole-induced dipole (DID) approximation. This approach is identical to that validated by our previous simulation for neat benzonitrile.^40^ A similar DID method was previously used by Ladanyi^41–45^ to simulate the acetonitrile/chloroform mixture, aromatic liquids and confined water in the OKE simulation and has been shown to accurately reproduce experimental OKE signals when combined with reliable force fields and *ab initio* polarizabilities. Based on a comparative study by Polok,^29^ the simple center–center DID approximation is sufficient to capture the median- and long-time orientational diffusion dynamics in hydrogen bonding liquids even though it may underestimate the fast librational motions (sub-picoseconds). Since our work focuses on the picosecond-to-nanosecond relaxation of acrylamide and water in mixtures rather than the sub-picosecond vibrational features, the DID model provides a reasonable and computationally efficient description of the collective polarizability anisotropy decay.

The total polarizability anisotropy time correlation function (PA-TCF), *ψ*_sum_(*t*), is then decomposed into three contributions as follows:

2 *ψ*_sum_(*t*) = *ψ*^MM^_sum_(*t*) + *ψ*^MI^_sum_(*t*) + *ψ*^II^_sum_(*t*)

This complete decomposition allows us to identify the relative importance of single-molecule reorientation (MM), interaction-induced effects (II), and their cross term (MI) as a function of concentration. The OKE nuclear response function is then obtained as follows:3
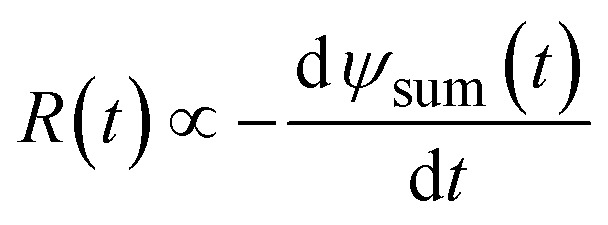


**Fig. 1:**
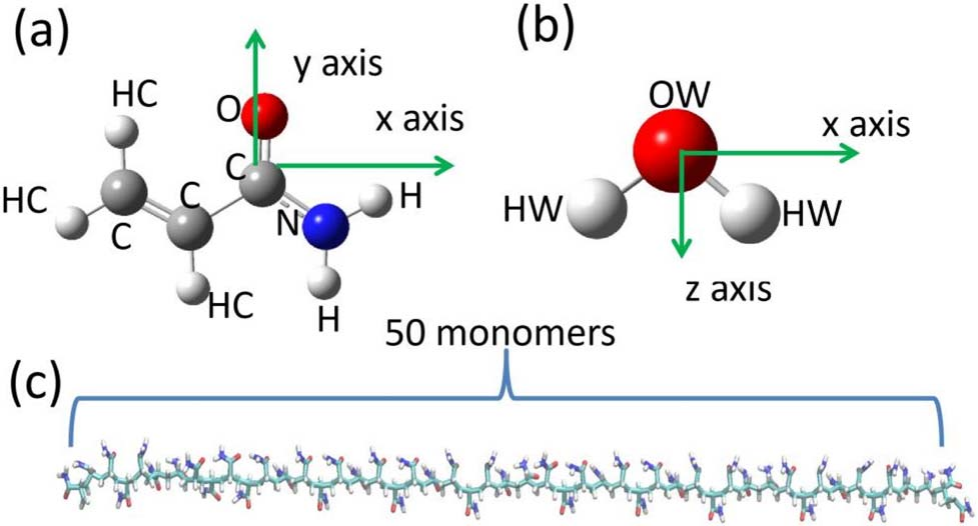
Molecular structures with the *x*, *y* and *z* axes of the molecular frame labeled. (a) Acrylamide (AAm); (b) water; and (c) polyacrylamide (PAAm).

For polyacrylamide (PAAm) solutions, the situation is fundamentally different. The polymer chain (50 repeat units, Fig. 1c) exhibits reorientational dynamics on a timescale of tens of nanoseconds (see SI Fig. S12), far exceeding the OKE experimental window (typically <100 ps). Therefore, its polarizability anisotropy does not relax on the measured timescale and contributes only as a static offset. As a result, we computed only the water contribution to the OKE response in the PAAm solutions, explicitly neglecting the polymer dynamics. This simplification is validated by the excellent agreement between our simulated water-only OKE transients and the experimental data of Van Wyck and Fayer.^21^ In contrast, for monomer (acrylamide) solutions, both solute and solvent contributions are included because acrylamide's rotational dynamics fall within the experimental time window (1–50 ps). For details on the relevant OKE response function calculations, please refer to the SI and our previous simulation article.^40^

### 2.2 Calculation and simulation details

The *ab initio* polarizabilities of acrylamide and water were calculated at the MP2/6-311++G(d,p) level using the Gaussian 09 package.^46^ The calculated principal components of the molecular polarizability of acrylamide are *α*_*xx*_ = 8.53 Å^3^, *α*_*yy*_ = 6.61 Å^3^, *α*_*zz*_ = 4.32 Å^3^, *α*_*xy*_ = −0.881 Å^3^, *α*_*yz*_ = 0.004 Å^3^, and *α*_*xz*_ = −0.005 Å^3^. The molecular polarizabilities of water are *α*_*xx*_ = 1.14 Å^3^, *α*_*yy*_ = 1.00 Å^3^, and *α*_*zz*_ = 1.17 Å^3^, which are very close to the data reported in previous OKE simulations of water.^47^ The structure of acrylamide is shown in Fig. 1a. The molecular coordinate axes for acrylamide are defined with the *y*-axis parallel to the C=O bond direction and the *z*-axis normal to the C–C–C–N chain plane. For the water molecule (Fig. 1b), the *y*-axis is defined as perpendicular to the water molecule plane, while the *z*-axis is defined as the direction of the bisector of the H–O–H angle.

MD simulations were performed using the OPLS-AA (acrylamide)^48^ and SPC/E (water) force fields^49^ at 300 K for acrylamide monomer/water mixtures and polyacrylamide aqueous solutions. Detailed information on the simulation systems is illustrated in Table S1 (SI). All MD simulations were performed using the Gromacs 2016 program.^50^ The simulations were initially equilibrated in the isothermal–isobaric ensemble (*NPT*) at 1 atm for 10 ns. The systems were then further equilibrated in a constant volume–temperature (*NVT*) ensemble. The monomer mixture system and polymer solution system were first equilibrated for 20 nanoseconds and 40 nanoseconds, respectively, followed by an additional 60 nanosecond production run in the *NVT* ensemble for each mixture system. The cross interactions between unlike atomic sites on different molecules were computed by a sum of coulomb and Lennard-Jones (6–12) terms with the Lorentz–Berthelot combination rules.^51^ In all simulations, the bond lengths were constrained using the LINCS algorithm.^52^ The cut-off for Lennard-Jones interactions was set to 12 Å. Long-range coulomb interactions were handled by the particle mesh Ewald (PME) with a cut-off of 12 Å and a grid spacing of 1.2 Å.^53^ The Berendsen thermostat^54^ was used in the NPT system to mimic the weak coupling between the system and a heat bath with a temperature coupling constant of 0.1 ps. In the *NVT* ensemble, a velocity rescaling thermostat^55^ was used for temperature coupling with a time coupling constant of 0.1 ps.

## 3. Results and discussions

### 3.1 OKE simulation spectroscopy and polarizability time correlation functions

**Fig. 2:**
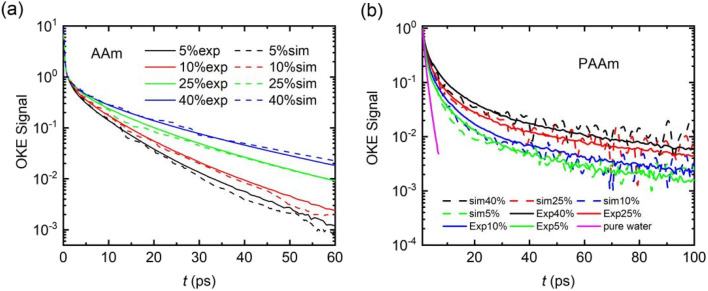
Comparison of the simulated and experimental OKE signals at different concentrations. (a) Acrylamide aqueous solutions. (b) Polyacrylamide aqueous solutions. The experimental data were obtained from Stephen Van Wyck and Michael Fayer.^21^

To validate the computational model and force field parameters, we first calculated the OKE response of AAm and PAAm aqueous solutions across a range of concentrations (5–40% w/v) and directly compared the results to the experimental measurements. Fig. 2 presents the simulated time-domain OKE transients for both systems. The excellent qualitative agreement with the experimental data of Van Wyck and Fayer^21^ demonstrates that our combination of force fields and the first-order DID polarizability approximation adequately captures the concentration-dependent dynamics. As clearly shown in Fig. 2, the OKE spectrum of the PAAm aqueous solution decays more slowly than that of the monomer aqueous solution; however, when its value drops to 0.01, it is still less than 100 ps. These decay dynamics are significantly faster than the reorientational dynamics of the polymer side chains and main chain; typically, the decay lifetimes of these chains are on the order of nanoseconds (see Fig. S12). In addition, the OKE decay dynamics of its aqueous solution are much slower than those of pure water, whereas pure water decays to the same value of 0.01 in less than 10 ps. This suggests that, for monomer aqueous solutions, the contribution of water to the overall OKE may not be significant, while for PAAm aqueous solutions, the contribution of the polymer chains to the total OKE may also not be decisive.

To carefully analyze the OKE dynamic relaxation processes, we fitted the OKE spectra of the AAm aqueous solution using a tri-exponential decay function and the OKE spectra of the PAAm aqueous solution using a bi-exponential decay function combined with a stretched exponential decay function: 
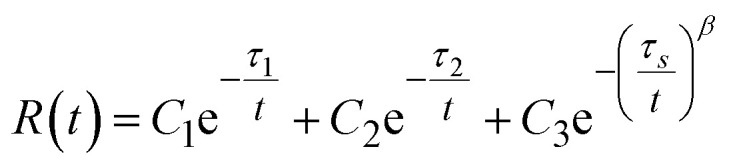
. We use the stretched exponential function form because previous OKE experiments and simulations^56,57^ on supercooled water have confirmed that its long-term decay dynamics exhibit a typical stretched exponential function form, consistent with MCT theory.^58^ All the relaxation times obtained from the fittings are listed in Table 1. For AAm monomer solutions, Van Wyck^21^ used a complex four-exponential decay function to fit the experimental OKE spectra, but such an overly complex function may result in overfitting. In fact, a tri-exponential decay function is sufficient to fit the current simulated OKE curves. From Table 1, the fastest relaxation component varies with concentration; it is significantly longer than that of bulk water, *i.e.* 0.16 ps, confirming that even the fastest decay in AAm solutions is dominated by the solute rather than water. The intermediate component, *τ*_2_, remains nearly constant at 5.3–5.4 ps for 5%, 10% and 25% but jumps to 10.81 ps at 40%. This abrupt increase indicates a possible change in the local environment at 40% w/v. Previous OKE experiments have suggested that the water content is insufficient to fully solvate every acrylamide monomer at the maximum concentration of 40%, leading to direct monomer–monomer contacts and the formation of small aggregates.^21^ Our calculations of the coordination numbers and the number of hydrogen bonds indicate that, at the highest concentration, aggregation between monomers becomes more pronounced, while the coordination numbers with water decrease significantly (see the following section, local structures). These aggregates reorient more slowly and hinder the rotational motion of nearby monomers. The slowest component, *τ*_3_, increases steadily from 12.36 ps (5%) to 31.20 ps (40%), reflecting the gradual slowing of overall acrylamide reorientation with increasing concentration and viscosity. Thus, the monomer OKE signal directly reports on the rotational dynamics of the acrylamide molecule itself, with water playing only a minor modulating role.

**Table 1:** Fitted relaxation time of the OKE spectrum in AAm and PAAm aqueous solutions^a^

	Concentration (w/v)								
	AAm 40%	AAm 25%	AAm 10%	AAm 5%	Pure water	PAAm 40%	PAAm 25%	PAAm 10%	PAAm 5%
*τ*_1_ (ps)	2.14	0.65	1.13	0.94	0.16	0.29	0.29	0.30	0.29
*τ*_2_ (ps)	10.81	5.45	5.30	5.36	2.47	3.35	2.87	3.02	2.58
*τ*_3_ (ps)	31.20	23.09	14.92	12.36					
*τ*_*s*_ (ps)						41.36	34.65	30.83	28.06
*β*						0.50	0.55	0.57	0.62

a The OKE for AAm solutions was fitted by triexponential decay functions. The bulk water was fitted by biexponential decay functions. The OKE for PAAm solutions was fitted by biexponential decay functions and single-stretched exponential functions, *i.e.*
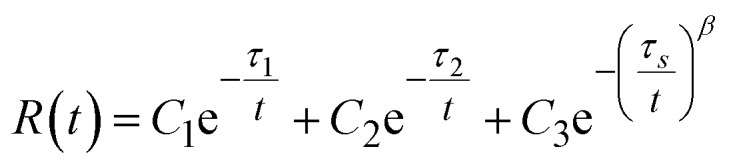
.

For PAAm polymer solutions, the OKE transients exhibit a fundamentally different behavior. The fastest component, *τ*_1_, is concentration-independent at ≈0.29 ps, which is very close to the pure water value 0.16 ps, and is assigned to fast water librations/hindered translations. The intermediate component, *τ*_2_, increases slightly from 2.58 ps (5%) to 3.35 ps (40%), indicating a moderate slowdown of water dynamics due to confinement. The slowest component is a stretched exponential with relaxation time *τ*_*s*_, which lengthens from 28.06 ps (5%) to 41.36 ps (40%), while the stretching exponent *β* decreases from 0.62 to 0.50. These *β* values are very close to the previous OKE spectral results for supercooled water.^56,57^ This stretched behavior is characteristic of heterogeneous, confined water dynamics, where water molecules experience a distribution of local environments within the polymer network. Crucially, no such slow-stretched component exists in monomer solutions. The polymer does not contribute to OKE decay because its reorientational motion occurs on a tens-of-nanoseconds timescale, far beyond the experimental window. Therefore, the OKE signal in PAAm solutions originates almost entirely from water molecules, whose dynamics are modulated by the polymer matrix.

**Fig. 3:**
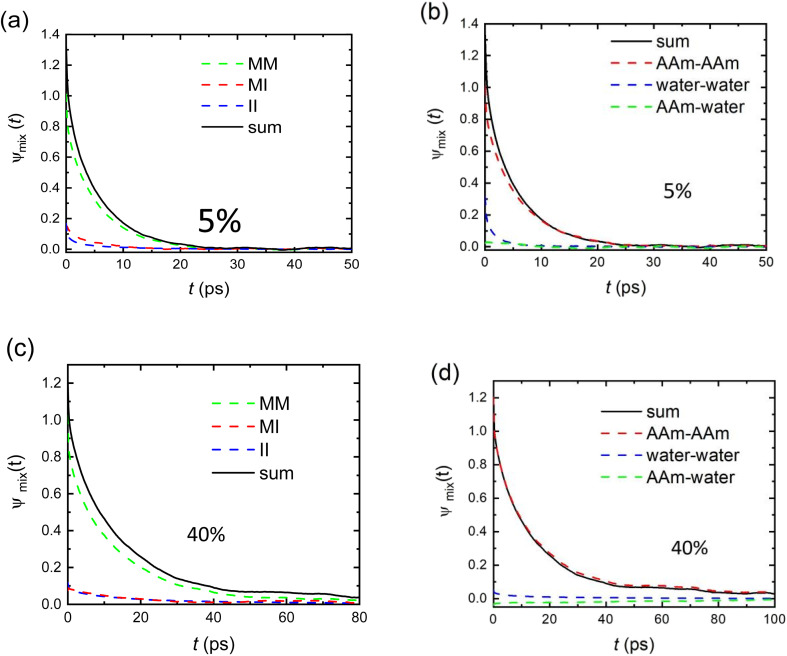
Polarizability anisotropy time correlation function (TCF) of acrylamide aqueous solutions. (a) Contributions of the molecule and interaction-induced autocorrelations and their cross-correlation at 5% w/v. (b) TCF of each component at 5% w/v. (c) Contributions of the molecule and interaction-induced autocorrelations and their cross-correlation at 40% w/v. (d) TCF of each component at 40% w/v.

To further analyze the contributions of the various components in the solution, we investigate their origins by decomposing the time-dependent function (TCF) of the anisotropy of the polarizability. Fig. 3 shows the decomposition for AAm solutions at 5% and 40% w/v. At both concentrations, the molecular (MM) part dominates the total TCF, while the interaction-induced (II) part is much smaller. This confirms that the OKE signal in monomer solutions arises primarily from single-molecule reorientation of acrylamide. At 40%, the II part becomes slightly more prominent, consistent with increased intermolecular coupling due to aggregation. The TCFs show that the total dynamics decay curves are dominated by the acrylamide part contribution, even at 5%. The water contribution is negligible.

**Fig. 4:**
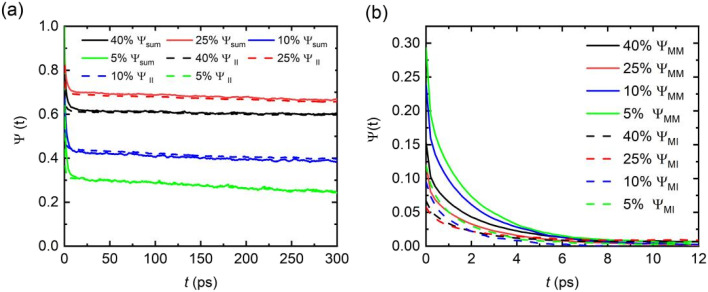
Polarizability anisotropy time correlation function (TCF) of polyacrylamide aqueous solutions. (a) Contributions of interaction-induced autocorrelations and total polarizability anisotropy TCF at different concentrations. (b) Contributions of the molecule and their cross-correlation TCF at different concentrations.

Fig. 4 presents the decomposition for PAAm solutions. Here, the situation is reversed: the II part is now approximately five times larger than the MM part and dominates the total TCF at all times. The MM part decays rapidly and contributes negligibly beyond a few picoseconds. This stark difference reflects the fact that water molecules in the polymer network are forced into correlated motions through collision-induced and cross-correlated interactions, whereas the polymer itself does not reorient on the measured time scale.

In summary, the combined analysis of the OKE spectrum and the TCF decompositions reveals a clean separation of dynamical origins: in AAm monomer solutions, OKE directly probes acrylamide rotational dynamics (dominated by MM); in PAAm polymer solutions, OKE probes water dynamics confined by the polymer network (dominated by II). Thus, while the monomer OKE signal directly reports on the reorientational dynamics of the acrylamide molecule, the polymer OKE signal provides an indirect, but highly sensitive, probe of the collective water dynamics and the water–polymer interaction-induced effects. The greatly enhanced II contribution in polymer solutions reflects the extensive and long-lived correlated motions induced by the polymer matrix, which acts to slow and couple the rotational and translational motions of the surrounding water molecules. This stark difference in the origin of the OKE response between monomer and polymer solutions highlights the need for careful decomposition of the polarizability TCF when interpreting experimental data from complex, crowded macromolecular systems.

### 3.2 Orientation dynamics and hydrogen bond dynamics

**Fig. 5:**
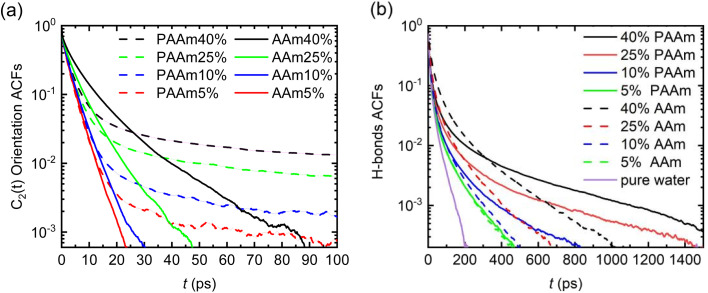
Orientation and hydrogen bonds (H bond) autocorrelation functions (ACFs) of water. (a) Orientation ACFs of the OW–HW bond direction in water at AAm and PAAm aqueous solutions. (b) H bond ACFs of OW–HW⋯OW between water molecules in AAm and PAAm aqueous solutions.

In aqueous solutions, hydrogen bonds between water molecules and orientational dynamics are key factors that determine the system's macroscopic diffusion properties and viscosity. Therefore, we first present the orientation autocorrelation functions (ACFs) of the OW–HW vector, as shown in Fig. 5a, for pure water, solutions of AAm monomer and solutions of PAAm polymer. The orientational ACF is defined as *C*_2_(*t*) = 〈*P*_2_(*û*(0)·*û*(*t*))〉, where *P*_2_ is the second Legendre polynomial and *û* is the unit vector along the OW–HW bond. This function reports on how quickly water molecules lose memory of their initial OW–HW bond direction. For pure water, ACF decay is rapid, with a fastest correlation time of approximately 1.0 ps, which is consistent with the well-known rapid reorientation dynamics of bulk water. For AAm monomer solutions, the decay process over short time scales is very similar to that of pure water, increasing only slightly with increasing concentration (*τ*_1_ ∼1.0–1.4 ps, see Table S2). This indicates that the presence of small mobile acrylamide monomers does not significantly hinder the fast rotational motion of water molecules. Water molecules can still rotate freely, and the hydrogen bond network remains highly dynamic. However, the orientational dynamics of the N–H bond in acrylamide closely resemble the OKE spectral decay lifetime of the monomer solution (Fig. S3). This observation aligns with the earlier TCF decomposition (Fig. 3), which shows that the OKE signal in monomer solutions is dominated by acrylamide, not by water.

For the PAAm polymer solution, the orientational ACF of the OW–HW bonds decays much more slowly, exhibiting a long tail. A significant fraction of the correlation persists beyond 50 ps, and the decay is not yet complete even at 100 ps. This long-tail effect of decay clearly indicates the presence of a locally heterogeneous environment. This directly reflects the confining effect of the polymer network. Water molecules form strong hydrogen bonds with the hydrophilic groups on the polymer, and their rotational motion is restricted by steric hindrance, which is also impeded by the need to break and reform hydrogen bonds within the confined geometric structure.

Fig. 5b shows the hydrogen bond (H bond) autocorrelation functions for water–water interactions, specifically for the OW–HW⋯OW hydrogen bond. The H bond ACF is defined as *C*_HB_(*t*) = 〈*h*(0)*h*(*t*)〉/〈*h*(0)〉, where *h*(*t*) = 1, if a given pair of water molecules is hydrogen-bonded at time *t* according to a geometric criterion (O–O distance <3.5 Å and O–H⋯O angle <30°) and 0 otherwise. Table 2 lists the tri-exponential fit parameters for the water–water hydrogen bond ACF (OW–HW⋯OW), together with the weighted average lifetime. For pure water, *τ*_average_ = 6.68 ps, representing the rapid making and breaking of hydrogen bonds characteristic of bulk water. For AAm monomer solutions, as the concentration increases, both the intermediate and the slowest components increase, while the fastest component remains small. For PAAm polymer solutions, the fast and intermediate relaxation components are nearly identical to those of the corresponding monomer solutions; the main difference lies in the slowest component. This observation is consistent with earlier two-dimensional infrared experiments by Roget *et al.*,^12^ which show that the structural lifetime and reorientation lifetime of water molecules remain virtually unchanged over short time scales, regardless of whether the system is a monomer solution, polymer solution, or hydrogel. However, we find that the slowest component in PAAm solutions is still much slower than that in AAm monomer solutions at high concentrations. This likely arises because the polymer chain has much slower structural relaxation compared to the monomer, which influences neighboring water molecules through hydrogen bonding and thereby modulates the slowest component among all water molecules. Meanwhile, for PAAm solutions, as the concentration increases, the amplitude coefficient of the slowest component decreases. This may be attributed to increased entanglement between polymer chains at high concentrations, which makes them more difficult to solvate than monomers, thus reducing the number of nearest-neighbor water molecules. Consequently, the hydrogen bond dynamics become extremely heterogeneous: a large fraction of water pairs experience very short hydrogen bond lifetimes (fast exchange), while a small fraction (≈3.4% of the pairs at 40% PAAm) is trapped in long-lived bonds with lifetimes approaching 200 ps.

**Table 2:** Fitted parameters of the OW–HW⋯OW hydrogen bonds by tri-exponential decay functions. *τ*_average_ is the weighted average lifetime, *τ*_average_ = *A*_1_*τ*_1_ + *A*_2_*τ*_2_ + *A*_3_*τ*_3_

w/v	*A*_1_	*τ*_1_ (ps)	*A*_2_	*τ*_2_ (ps)	*A*_3_	*τ*_3_ (ps)	*τ*_average_ (ps)
Pure water	0.19	0.32	0.63	4.53	0.17	22.03	6.68
5% AAM	0.23	0.66	0.62	6.02	0.12	34.76	7.97
10% AAM	0.24	0.85	0.60	6.85	0.12	39.56	8.91
25% AAM	0.27	1.49	0.55	10.34	0.12	58.52	12.89
40% AAM	0.31	3.24	0.48	18.94	0.11	103.52	21.05
5% PAAM	0.24	0.64	0.62	5.72	0.11	34.60	7.62
10% PAAM	0.29	1.08	0.57	6.95	0.09	47.18	8.37
25% PAAM	0.43	2.18	0.43	10.94	0.05	87.86	10.39
40% PAAM	0.54	3.21	0.31	17.56	0.03	192.70	13.78

**Fig. 6:**
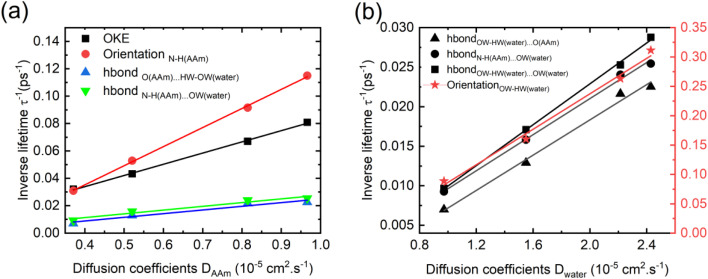
Linear relationship between the diffusion coefficients of components and the inverse lifetime of OKE and orientation and h bond dynamics in AAm aqueous solutions. (a) Linear trends of the AAm diffusion coefficients and the inverse lifetime of the OKE, N–H orientation, and hydrogen bonds between water and AAm. (b) Linear trends of the water diffusion coefficients and the inverse lifetimes of the OW–HW orientational dynamics and hydrogen bond dynamics within water and AAm. The lifetime of these orientational and h bond dynamics were from the slowest relaxation time of the triexponential decay functions fitted by the corresponding dynamical decay curves.

Fig. 6a plots the self-diffusion coefficient of acrylamide *D*_AAm_ against four inverse lifetimes obtained from tri-exponential fits to the OKE transients, the N–H bond reorientational correlation functions, and two hydrogen bond dynamics between acrylamide and water. The self-diffusion coefficients of acrylamide were calculated from the mean-squared displacement of the acrylamide center-of-mass over time using the Einstein relation. The inverse lifetimes (1/*τ*) are taken from the slowest component of the tri-exponential fits because this component dominates the long-time relaxation and is directly related to the rotational diffusion of the acrylamide molecule.

These dynamical behaviors also exhibit a good linear relationship with the macroscopic diffusion coefficient of the AAm molecules, fully demonstrating that these dynamics are coupled with each other. The OKE spectrum is also directly related to the monomer molecular diffusion dynamics, once again proving unequivocally that the long-time OKE decay is controlled by the rotational diffusion of the acrylamide molecule. This conclusion is fully consistent with the TCF decomposition, as depicted in Fig. 3, where the molecular (MM) part dominates the total polarizability anisotropy. However, the slowest relaxation lifetimes of the two types of hydrogen bonds between the monomer and water are almost identical, indicating that the hydrogen bond strengths between the C=O group and water and between the N–H group and water are very similar. As illustrated in Fig. 6b, the self-diffusion coefficients of water are directly linearly correlated with the rotational and hydrogen bond dynamics of water molecules, as well as with the hydrogen bonds between water and AAm monomer molecules. All the linear trends shown in Fig. 6 clearly demonstrate such a physical transport mechanism: the hydrogen bond dynamics between water and AAm completely regulate the transport behavior of both water molecules and AAm monomer molecules, which couples the translational and rotational dynamics of the molecules, thus exhibiting good hydrodynamic behavior. The hydrodynamics model can also be validated using the Stokes–Einstein–Debye equation (see Fig. S9). This synchronization can be understood as follows: acrylamide molecules, with their polar amide groups, form a transient hydrogen bond network that connects multiple water molecules. When a water molecule diffuses translationally, it must break its existing hydrogen bonds (both with other water molecules and with acrylamide) and form new ones. The rate-limiting step for both translation and rotation is the same—namely, the rearrangement of the hydrogen bond network. Therefore, all dynamical observables become proportional to each other. This behavior is reminiscent of “strongly coupled” dynamics found in some hydrogen bond liquids near the glass transition, where the decoupling between rotation and translation disappears due to cooperative motion.

**Fig. 7:**
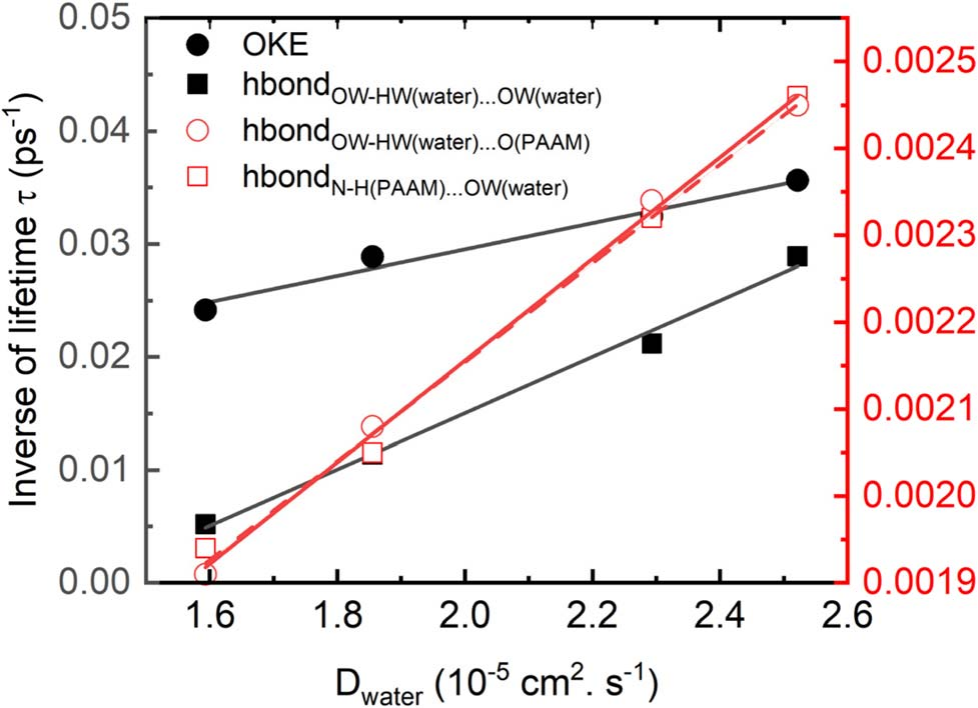
Linear relationships of the inverse lifetimes for OKE and hydrogen bonds with the diffusion coefficient of water in PAAm aqueous solutions.

For PAAm polymer solutions, the OKE signal originates almost exclusively from water because the polymer chain reorients on a tens-of-nanoseconds timescale, far beyond the OKE experimental window (≤100 ps). The slowest OKE decay is fitted by a stretched exponential with relaxation time *τ*_*s*_ and stretching exponent *β* (see Table 2). Fig. 7 plots *D*_water_ against 1/*τ*_*s*_ and against the inverse lifetimes of the water–water hydrogen bond and the two types of polymer–water hydrogen bonds. All these inverse lifetimes are strictly proportional to *D*_water_ across the entire concentration range. This indicates that the macroscopic translational mobility of water in the polymer network is governed by the same microscopic process that determines the OKE signal—namely, the restructuring of the water hydrogen bond network under confinement.

Crucially, the orientational dynamics of the water OW–HW bond behave differently in PAAm solutions. As shown in Fig. S13, the weighted average inverse lifetime of the OW–HW orientation, 1/*τ*_average_, scales linearly with *D*_water_. However, the inverse of the slowest component, 1/*τ*_3_, does not exhibit a linear relationship with *D*_water_. This finding means that while the overall (average) rotational rate of water is coupled to translation, the slowest rotational relaxation process becomes decoupled. This decoupling is characteristic of confined and supercooled water,^56–59^ where the hydrogen bond network becomes more rigid and cooperative. The stretched exponential parameter *β* decreases from 0.62 at 5% to 0.50 at 40% (Table 2), further supporting a wide distribution of relaxation times and heterogeneous dynamics. This behavior is reminiscent of the structural relaxation observed in supercooled water, where a *β* value around 0.6 is a signature of fragile glass-forming liquids.^56,57^ Similarly, MD simulations of water confined in nanoporous silica have revealed a universal stretched exponential relaxation with *β* ≈ 0.6, attributed to dynamic heterogeneities induced by the confining environment.^58^ Furthermore, studies on water confined in reverse micelles have highlighted significant dynamic heterogeneity, where water dynamics are spatially stratified based on the distance from the hydrophilic interface.^59^ The even lower *β* values observed here suggest that the PAAm network imposes a higher degree of dynamic heterogeneity and cooperativity on the hydration water. This behavior is not observed in monomer solutions, where both the average and slowest rotational rates remain coupled to translation.

Additionally, the rates of the two types of polymer–water hydrogen bonds are nearly identical to the water–water hydrogen bond rate at a given concentration. This implies that water molecules in the hydration shell of PAAm exchange rapidly with bulk-like water and that the polymer does not create a distinct, kinetically separated water population. Instead, the entire water hydrogen bond network is uniformly slowed down by the presence of the polymer chains, which act as additional hydrogen bonding sites that increase the effective network connectivity and free energy barriers for bond exchange.

Finally, it is important to reiterate the role of the polymer chain. The reorientational dynamics of the PAAm polymer chain (both main chain and side chains) occur on a time scale of nanoseconds or longer, as estimated from the rotational diffusion coefficient of a 50-mer chain. This is far beyond the 100 ps time window of our OKE experiment. Therefore, the OKE signal contains no contribution from polymer reorientation; the MM part of the polarizability TCF is essentially static on the experimental timescale. The measured decay is entirely due to the II part, which arises from the motion of water molecules relative to the polymer and from water–water interactions.

However, the polymer chain indirectly influences water diffusion through its confining effect. The polymer matrix acts as a dynamic hydrogen bond cage: water molecules diffuse through the winding network, where the breaking of hydrogen bonds is the rate-limiting step. It is precisely the confined hydrogen bond dynamics of water on the polymer surface and within the shell that cause the overall slowing of the diffusion kinetics of the confined water. Therefore, although the motion of the polymer network is too slow to be directly observed in the OKE experiments, the diffusion coefficient of water is still determined by the aggregation structure of the polymer network and the hydrogen bonding dynamics with water. This indirect coupling relationship is reflected in the linear correlation shown in Fig. 7.

### 3.3 Local structures

Changes in dynamic properties are typically caused by structural changes. To further elucidate the mechanisms underlying these dynamic properties, we calculated various radial distribution functions (RDFs) and hydrogen-bond numbers in both AAm and PAAm solutions. Based on the structural analysis, we can now establish a comprehensive picture linking local structure, hydrogen-bond populations, and dynamical behavior.

**Fig. 8:**
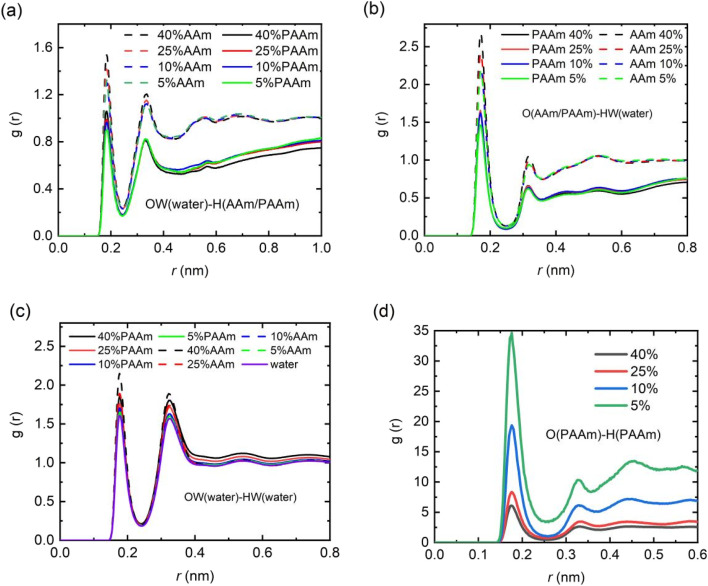
Site–site radial distribution functions in AAm and PAAm aqueous solutions. (a) OW–H (AAm/PAAm). (b) O (AAm/PAAm)–HW (water). (c) OW (water)–HW (water). (d) O (PAAm)–HN (PAAm).

Fig. 8 presents the site–site RDFs for key atomic pairs in AAm and PAAm aqueous solutions at different concentrations. Fig. 8a shows the RDF between water oxygen (OW) and the amide hydrogen (H) of the solute (AAm or PAAm). For AAm solutions, the first peak appears at ≈1.95 Å with a high intensity, and the peak height decreases systematically with increasing concentration. This indicates that as the monomer concentration rises, fewer water molecules occupy the first solvation shell around the N–H group. For PAAm solutions, the peak position is nearly identical, but the intensity is noticeably lower than that of AAm at the same concentration. This suggests that in the polymer, the N–H groups are partially shielded by chain crowding, leading to reduced water accessibility. Fig. 8b displays the RDF between the solute carbonyl oxygen (O) and water hydrogen (HW). For AAm, a strong first peak is observed at ≈1.80 Å, with the peak height decreasing as concentration increases. For PAAm, the peak position remains similar, but the intensity is again lower than that of AAm at comparable concentrations. This indicates that the carbonyl oxygen in the polymer is also less accessible to water, consistent with chain entanglement and steric hindrance.

Fig. 8c shows the RDF for water–water interactions (OW–HW⋯OW). For both the AAm and PAAm solutions, the first peak appears at ≈1.75 Å, and the peak position does not shift significantly with concentration or solute type. However, the peak height for PAAm solutions is consistently higher than that for AAm at the same concentration, especially at high concentrations (40%). This implies that water–water correlations are stronger in the polymer solutions, suggesting that water molecules form a more structured and persistent network when confined by the polymer matrix.

Fig. 8d presents the RDF between the polymer carbonyl oxygen (O) and the amide hydrogen (HN) of PAAm itself (*i.e.*, intra- or inter-chain amide–amide interactions). For PAAm, a distinct peak appears at ≈2.25 Å, which is much longer than typical hydrogen-bond distances (≈1.8–2.0 Å). This indicates that the O⋯HN contacts are primarily coulombic (electrostatic) rather than hydrogen-bonded, reflecting the strong dipole–dipole interactions between amide groups along the polymer chains.

To quantify these structural features, we calculated the coordination numbers (CNs) from the first shell of each RDF, as listed in Table 3. For the OW–HW RDF, the CN decreases from 1.85 (5% AAm) to 1.38 (40% AAm), while for PAAm, it decreases from 1.84 to 1.73 over the same concentration range. This shows that water–water coordination is more suppressed in monomer solutions than in polymer solutions, consistent with the higher water–water RDF peak heights observed for PAAm. For the OW–H CN, the AAm values decrease from 0.88 (5%) to 0.68 (40%), whereas for PAAm, they remain relatively constant. This indicates that in polymer solutions, the number of water molecules near the amide hydrogen does not decrease sharply with concentration because the polymer chain provides a persistent but less accessible surface. For the O–HW CN, AAm shows a decrease from 2.25 (5%) to 1.68 (40%), while PAAm shows a smaller decrease from 1.96 to 1.75. This again confirms that the polymer carbonyl groups are less solvated than monomers, but the reduction with concentration is milder. Finally, for the O–H CN, AAm has very low values (0.04–0.40), reflecting negligible monomer–monomer contacts, while PAAm exhibits much higher values (0.52–0.57), indicating significant inter- or intra-chain amide–amide coulombic interactions, consistent with the RDF peak, as depicted in Fig. 8d.

**Table 3:** Coordination number (CN) of the site–site radial distribution functions

w/v	*N*_OW(water)–HW(water)_	*N*_H(AAm/PAAm)–OW(water)_	*N*_O(AAm/PAAm)–HW(water)_	*N*_O(AAm/PAAm)–H(AAm/PAAm)_
40% AAm	1.38	0.68	1.68	0.40
25% AAm	1.71	0.76	1.97	0.23
10% AAm	1.82	0.85	2.18	0.10
5% AAm	1.85	0.88	2.25	0.04
40% PAAm	1.73	0.57	1.75	0.57
25% PAAm	1.78	0.59	1.84	0.56
10% PAAm	1.82	0.60	1.94	0.54
5% PAAm	1.84	0.62	1.96	0.52

Table 4 lists the hydrogen-bond numbers for all types of bonds. For AAm solutions, the water–water hydrogen-bond count (OW–HW⋯OW) decreases from 1.765 (5%) to 1.484 (40%), while the total AAm–water hydrogen bonds increase from 3.350 to 4.328. For PAAm solutions, the water–water hydrogen-bond count remains higher, while the total polymer–water hydrogen bonds are much lower (2.489 at 40% for PAAm *vs.* 3.350 for AAm). This clearly demonstrates that in PAAm, the amide groups are less efficiently solvated by water, forcing water molecules to maintain more water–water hydrogen bonds. The pure water value for OW–HW⋯OW is 1.796, showing that both solutes reduce water–water hydrogen bonding, but the reduction is significantly stronger in monomer solutions.

**Table 4:** Hydrogen bond numbers in the AAm and PAAm aqueous solutions^a^

Number of hydrogen bonds *N*	Concentration (w/v)							
	AAm 40%	AAm 25%	AAm 10%	AAm 5%	PAAm 40%	PAAm 25%	PAAm 10%	PAAm 5%
*N*_OW–HW⋯OW_	1.484	1.621	1.733	1.765	1.668	1.712	1.760	1.779
*N*_OW–HW⋯O(AAm/PAAm)_	1.680	1.910	2.037	2.108	1.240	1.337	1.351	1.371
*N*_N–HN(AAm/PAAm)⋯OW_	1.670	1.874	2.125	2.220	1.249	1.164	1.238	1.248
*N*_Total AAm/PAAm–water_	3.350	3.784	4.162	4.328	2.489	2.501	2.589	2.619

a The number of OW–HW⋯OW hydrogen bonding for pure water in our simulation is 1.796.

The structural data explain the dynamical dichotomy observed earlier. In monomer solutions, the high density of accessible amide groups creates a “water-bridged” solvation shell where water molecules are strongly bound to acrylamide, reducing the water–water hydrogen-bond population but not drastically slowing down the remaining water network because monomers are small and mobile. Water dynamics are governed primarily by the exchange between bulk-like water and the monomer hydration shell, which occurs on a faster timescale. In polymer solutions, the amide groups are partially buried, and the long polymer chains create steric hindrance that restricts water motion. The water–water hydrogen-bond count remains high, but these water molecules are confined within the mesh of the polymer network. This confinement slows down both water diffusion and hydrogen-bond exchange, as evidenced by the linear correlation between *D*_water_ and water–water hydrogen-bond dynamics (see Fig. 7). Moreover, the polymer–water hydrogen-bond dynamics are proportional to *D*_water_, indicating that water molecules that form hydrogen bonds with the polymer are in rapid exchange with the bulk-like water population and that the entire water network experiences a uniform slowdown due to confinement. The stretched exponential parameter *β* ≈ 0.6 in the OKE decay of polymer solutions further supports a heterogeneous environment with a wide distribution of relaxation times, characteristic of confined water. This contrasts with monomer solutions, where the OKE decay is multi-exponential with a dominant component from acrylamide rotation. The structural analysis thus provides a solid foundation for understanding the distinct dynamical behaviors observed in monomer *versus* polymer aqueous solutions.

The water–water hydrogen bond number is higher in PAAm than in AAm at the same concentration, yet the water dynamics are slower in PAAm. This indicates that confinement, not just the number of hydrogen bonds, controls dynamics. The retained water network is more rigid due to spatial restrictions by polymer chains.

Solute–water hydrogen bond number is lower in PAAm than in AAm, suggesting that polymer chain folding reduces effective hydrogen bonding sites. However, the kinetics of these bonds are directly proportional to water diffusion, implying that polymer–water hydrogen bonds are not kinetically independent but rather part of the same dynamical network.

## 4. Conclusions

In this work, we performed extensive molecular dynamics simulations to investigate the dynamics of AAm and PAAm aqueous solutions using OKE spectroscopy as a reference. The simulated OKE transients, calculated using the first-order dipole-induced dipole (DID) polarizability approximation, are in excellent qualitative agreement with the experimental measurements across a wide concentration range. Through systematic decomposition of the polarizability anisotropy time correlation function into its molecular (MM), interaction-induced (II) and cross (MI) contributions, we elucidated the fundamentally different origins of the OKE signal in monomer *versus* polymer solutions. For AAm monomer solutions, the MM contribution overwhelmingly dominates the total TCF at all concentrations, confirming that the OKE response directly probes the rotational diffusion of the acrylamide molecule. The slowest relaxation time (*τ*_3_) increases steadily, reflecting the concentration-dependent slowing of solute reorientation. The abrupt increase in *τ*_2_ at 40% provides clear evidence of incomplete solvation and the onset of monomer aggregation at the highest concentration.

For PAAm polymer solutions, the situation is reversed: the II contribution dominates the total TCF and is approximately five times larger than the MM part. The polymer chain does not contribute to OKE decay because its reorientational motion occurs on a tens-of-nanoseconds timescale, far beyond the experimental window. Instead, the OKE signal arises entirely from water molecules, whose dynamics are confined by the polymer network. The slowest relaxation is described by a stretched exponential with *τ*_*s*_ increasing from 28.06 ps at 5% to 41.36 ps at 40% and stretching exponent *β* decreasing from 0.62 to 0.50, indicating increasingly heterogeneous dynamics at higher polymer concentrations. This stretched behavior is the characteristic of confined water and is absent in monomer solutions.

The linear correlations established between diffusion coefficients and the inverse lifetimes of OKE, orientational, and hydrogen bond dynamics demonstrate that translational diffusion, rotational relaxation, and hydrogen bond exchange are strongly coupled in both monomer and polymer solutions. For monomer solutions, all dynamical observables scale identically with water diffusion, indicating that the hydrogen bond network synchronizes these processes. For polymer solutions, although the OKE and hydrogen bond dynamics scale linearly with water diffusion, the slowest component of water rotation becomes decoupled from translation, a hallmark of confinement effects.

Our results demonstrate that the simple center–center DID approximation, despite its known limitations in capturing sub-picosecond librational motions, provides a reasonable and computationally efficient description of the picosecond-to-nanosecond orientational dynamics in hydrogen bonding systems. The methodology developed herein—combining MD simulations, DID polarizability calculations, and TCF decomposition—offers a general framework for interpreting OKE spectra from complex molecular systems, including polymer solutions, hydrogels, and other soft materials. Future studies will extend this approach to cross-linked hydrogels and more complex macromolecular architectures, where the interplay between polymer network dynamics and confined water motion plays a crucial role in determining macroscopic transport properties. Additionally, the application of more sophisticated polarizability models, such as the atomic DID (ADID) or pre-optimized atomic DID (OADID) methods, may further improve the accuracy of short-time dynamics while maintaining the computational efficiency required for large-scale simulations.

## Author contributions

The manuscript was written through contributions from all the authors.

## Conflicts of interest

The authors declare that there are no competing financial interests.

## Supplementary Material

RA-OLF-D6RA05577J-s001

## Data Availability

All the data for this study are provided in the manuscript as well as in the supplementary information (SI). Supplementary information: simulation details, OKE polarizability correlation functions, orientational dynamics, hydrogen bond dynamics, diffusion coefficients, validation of the hydrodynamic model. See DOI: https://doi.org/10.1039/d6ra05577j.
