# Supplementary material for: Dynamics and microstructures in aqueous solutions of polyacrylamide and acrylamide monomers assessed by molecular dynamics simulations combined with optical Kerr effect spectroscopy
Source: RSC Adv. 2026 Aug 17;16(41):48364–76. doi: 10.1039/d6ra05577j (PMC13479955; doi:10.1039/d6ra05577j)
Supplement: Supplementary file 1 [file RA-016-D6RA05577J-s001.pdf]

Supplementary Information (SI) for RSC Advances.
This journal is © The Royal Society of Chemistry 2026

Supplementary Information

Dynamics and Microstructures in Aqueous Solutions of Polyacrylamide and Acrylamide Monomers by Molecular Dynamics Simulation in Contrast to Optical Kerr Effect Spectroscope

Lin Wang [†], Huiqin Wang [†] and Maolin Sha*, [†]

[†]Department of Physics and Materials Engineering
Hefei Normal University, Hefei 230061, China
Email: mlsha@hfnu.edu.cn

1. Detail of simulation systems

Table S1. The MD simulation systems and run time

Concentration (w/v)	N_{AAm}/N_{PAAm}	N_{water}	*Box (nm³)*	*Run time (ns)*	*Density(g/cm³)*
AAm 40%	300	1800	84.40	80	1.058
AAm 25%	200	2400	93.28	80	1.027
AAm 10%	100	3600	118.54	80	1.008
AAm 5%	50	3750	118.47	80	0.997
PAAm 40%	8	3948	146.24	100	1.131
PAAm 25%	4	3159	108.53	100	1.088
PAAm 10%	2	3909	125.01	100	1.030
PAAm 5%	1	3748	116.50	100	1.013

2. OKE Theoretical Background

In a binary mixture consisting of two distinct molecular species u (acrylamide) and v (water), the collective polarizability tensor Π_{sum} is given by the sum of molecular and interaction-induced contributions from both components:

$$\Pi_{sum} = \Pi^M + \Pi^I = (\Pi_u^M + \Pi_v^M) + (\Pi_u^I + \Pi_v^I) \tag{S1}$$

The molecular part is the sum of individual molecular polarizabilities:

$$\Pi_u^M = \sum_{i=1}^{N_u} \alpha_i^0, \quad \Pi_v^M = \sum_{i=1}^{N_v} \alpha_i^0 \tag{S2}$$

The interaction-induced part arises from dipole-induced dipole (DID) interactions. Within the first-order DID approximation, it is expressed as:

$$\Pi_u^I = \sum_{i=1}^{N_u}\sum_{j \neq i}^{N_u} \alpha_i^0 \cdot T_{ij} \cdot \alpha_j^0 + \sum_{i=1}^{N_u}\sum_{j=1}^{N_v} \alpha_i^0 \cdot T_{ij} \cdot \alpha_j^0 \tag{S3}$$

and analogously for Π_v^I, where $T_{ij} = (3\hat{r}_{ij}\hat{r}_{ij} - I)/r_{ij}^3$ is the dipole interaction tensor.

The polarizability anisotropy time correlation function (PA-TCF) for the mixture is defined as:

$$\psi_{sum}(t) = \frac{\langle \Pi_{XY}(0)\Pi_{XY}(t) \rangle}{\Gamma^2} \tag{S4}$$

where $\Gamma^2 = N_u\gamma_u^2/15 + N_v\gamma_v^2/15$ is the ideal integrated intensity, with γ^2 being the squared molecular polarizability anisotropy $\gamma^2 = x_u\gamma_u^2 + x_v\gamma_v^2$. Following the decomposition in the collective polarizability tensor of Equation 1, the total PA-TCF separates into three contributions:

$$\psi_{sum}(t) = \psi_{sum}^{MM}(t) + \psi_{sum}^{MI}(t) + \psi_{sum}^{II}(t) \tag{S5}$$

Each term further decomposes into intra-species and cross-species parts, e.g.:

$$\psi_{sum}^{MM}(t) = \frac{1}{\Gamma^2}\left[N_u\gamma_u^2\psi_u^{MM}(t) + N_v\gamma_v^2\psi_v^{MM}(t) + 2\sqrt{N_uN_v}\gamma_u\gamma_v\psi_{uv}^{MM}(t)\right] \tag{S6}$$

where $\psi_u^{MM}(t)$ is the normalized molecular autocorrelation for species u, and $\psi_{uv}^{MM}(t)$ is the cross-correlation between different species. In the present work, we do not employ the projected representation (i.e., we do not separate ψ_{RR} from collision-induced

components); instead we compute the three ψ_{MM}, ψ_{MI}, and ψ_{II} directly from MD trajectories. This complete decomposition allows us to identify the relative importance of single-molecule reorientation (MM), interaction-induced effects (II), and their cross term (MI) as a function of concentration. The OKE nuclear response function is then obtained as

$$R(t) \propto -\frac{d\psi_{sum}(t)}{dt} \tag{S7}$$

3. The time correlation function (TCF) for AAm/water mixture.

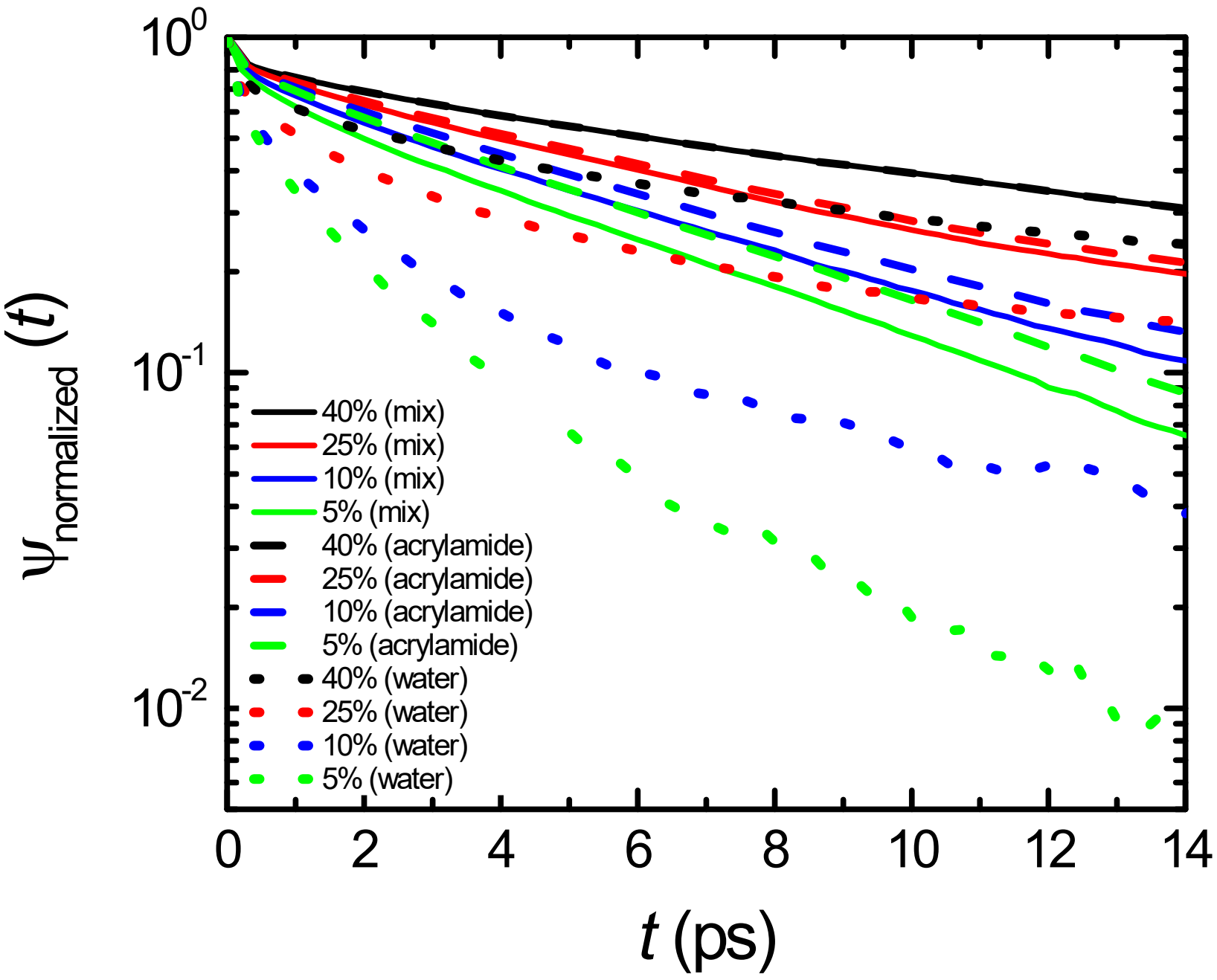

Figure S1. The time correlation function (TCF) for AAm/water mixture and each component.

4. The contributions of the molecule and interaction-induced autocorrelations and their cross-correlation for polarizability anisotropy time correlation function (TCF) .

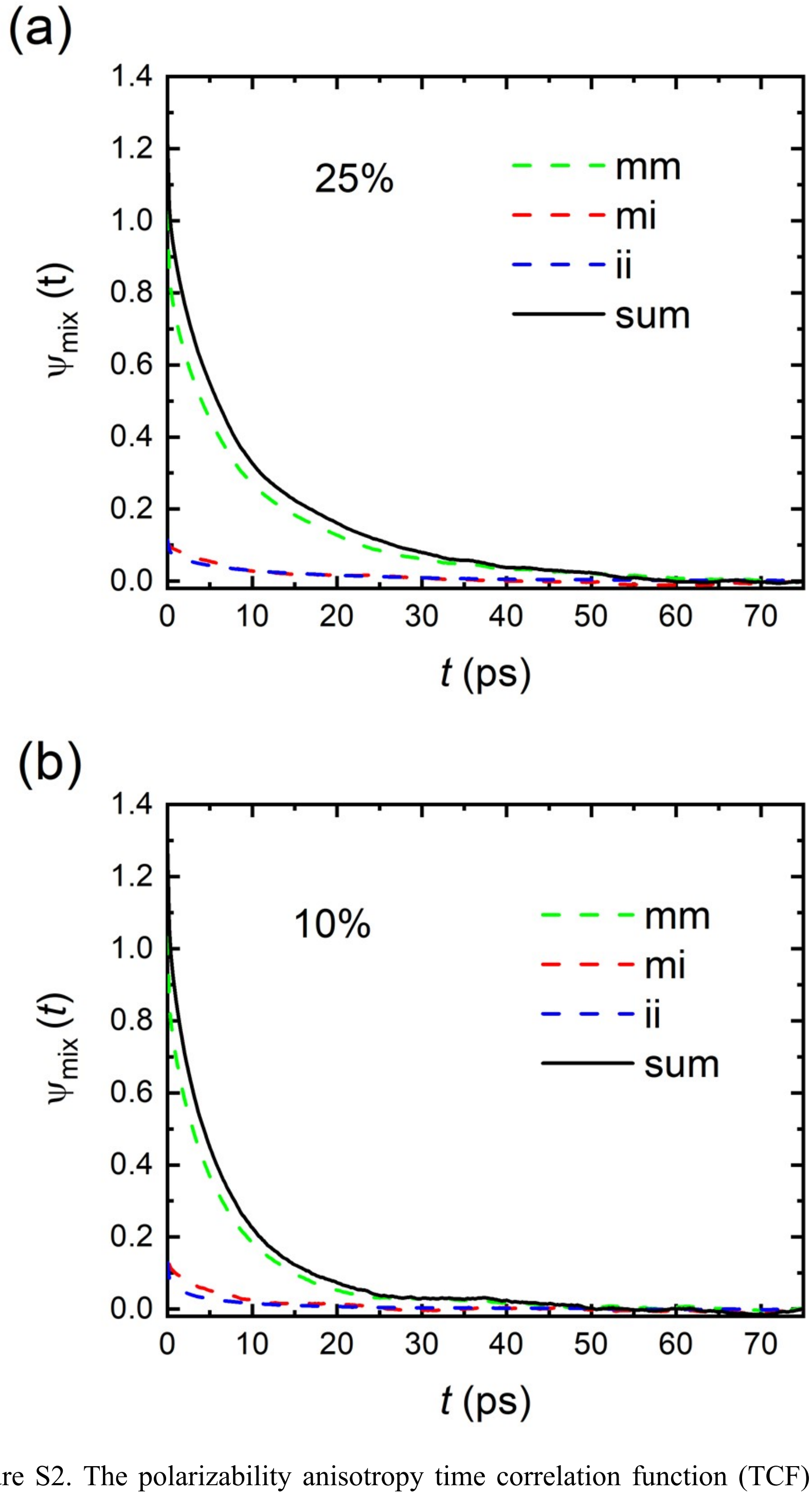

Figure S2. The polarizability anisotropy time correlation function (TCF) of acrylamide aqueous solutions. (a) The contributions of the molecule and interaction-induced autocorrelations and their

cross-correlation at 25% w/v. (b) The contributions of the molecule and interaction-induced autocorrelations and their cross-correlation at 10% w/v.

5. The orientational auto-correlation functions of N-H bond in AAm aqueous solutions.

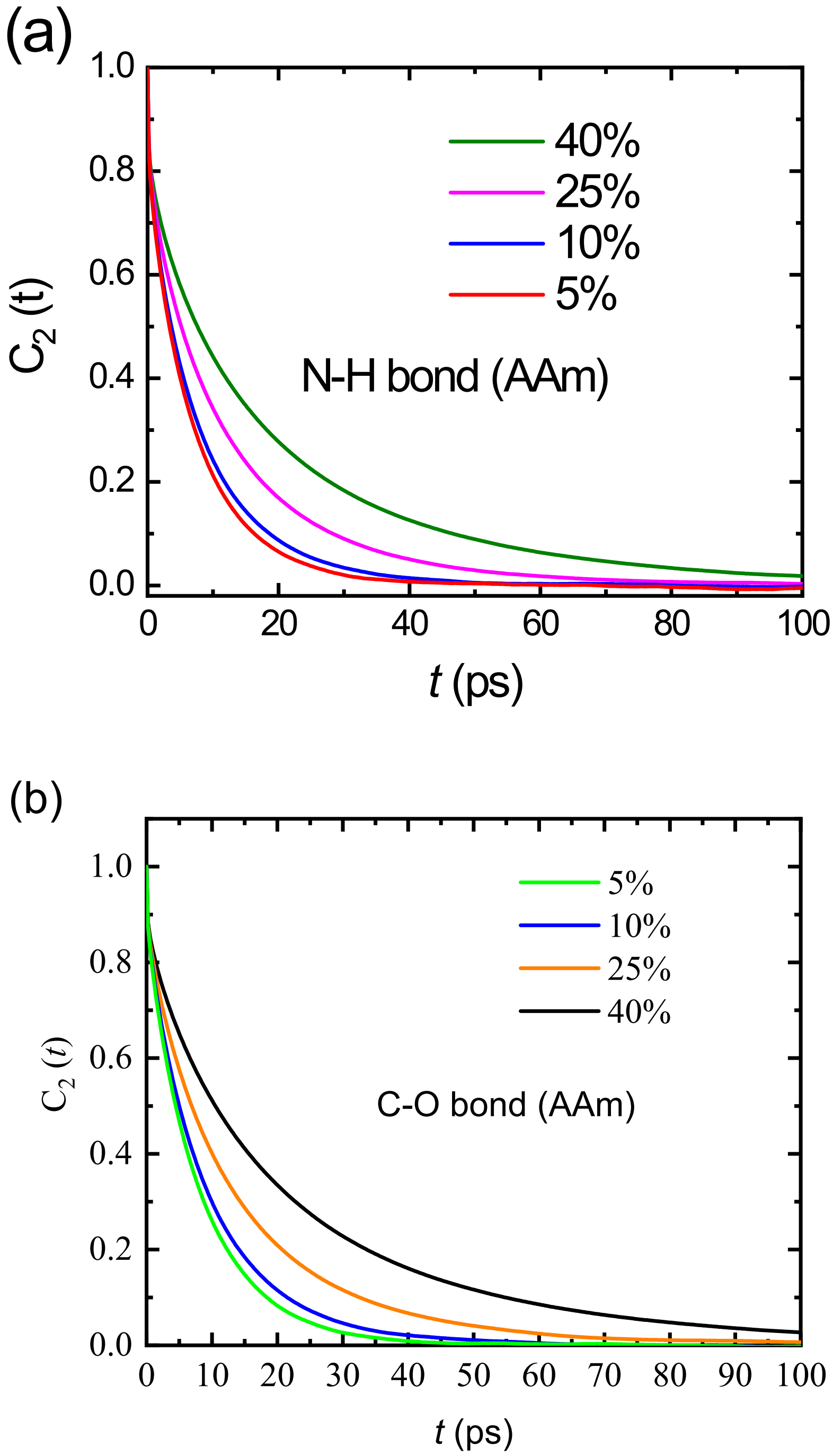

Figure S3. The orientational auto-correlation functions for AAm aqueous solutions. (a) O-H bond. (b) C-O bond.

6. The hydrogen bond dynamics between AAm and water for AAm aqueous solutions

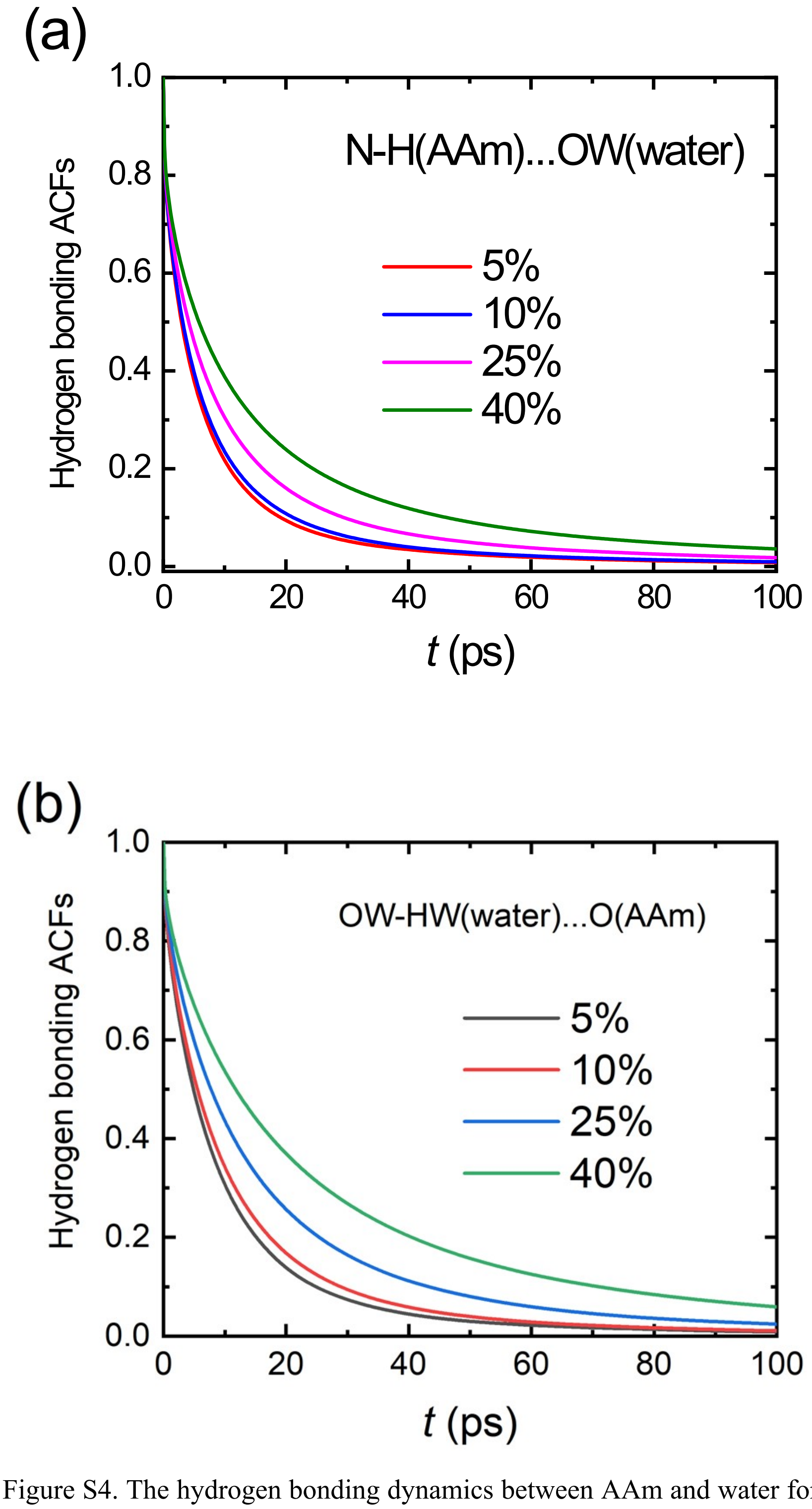

Figure S4. The hydrogen bonding dynamics between AAm and water for AAm aqueous solutions. (a) N-H(AAm)...OW(water) (b) OW-HW(water)...O(AAm)

7. The interaction energies in AAm aqueous solutions

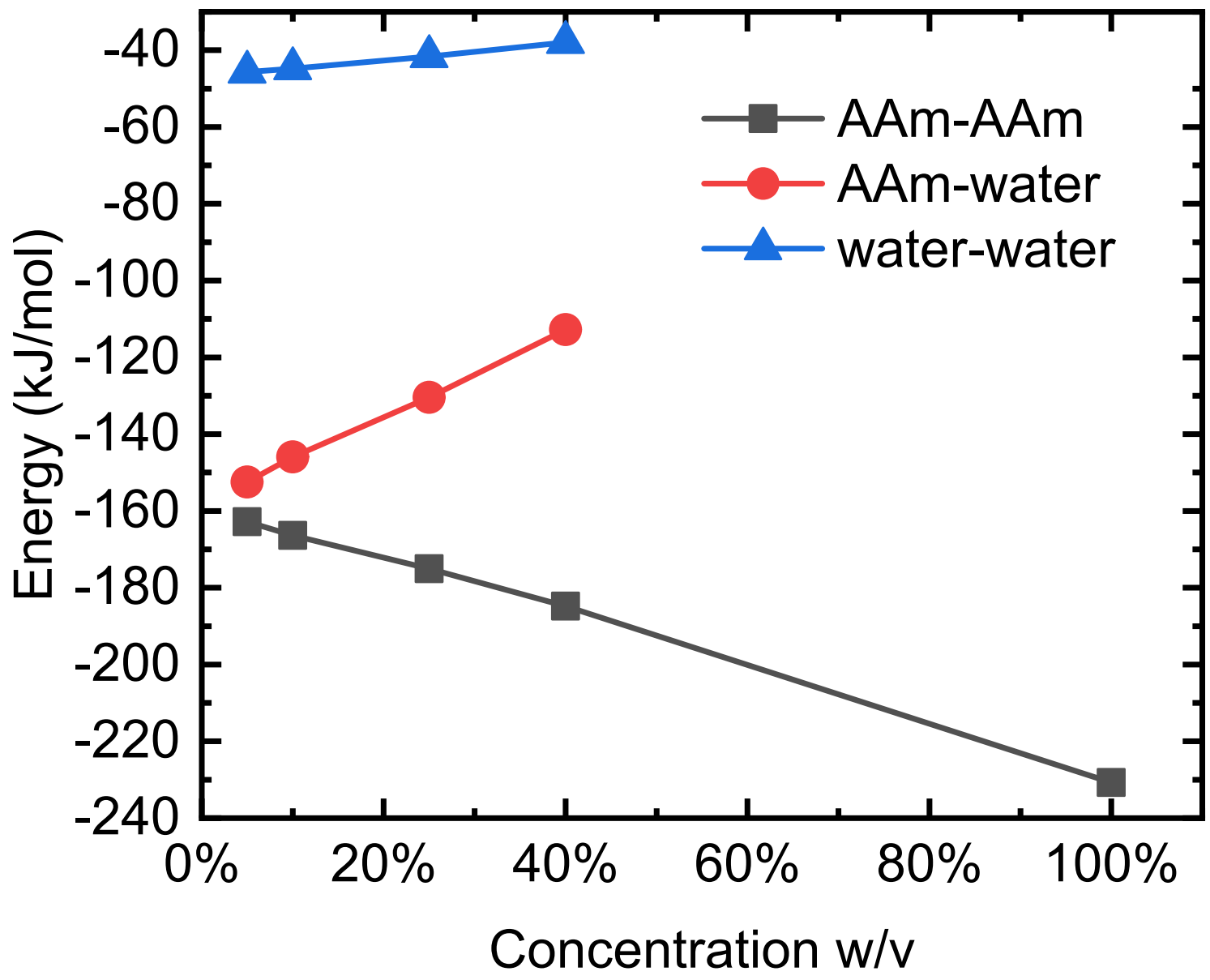

Figure S5. The interaction energies in AAm aqueous solutions.

8. The self-diffusion coefficients

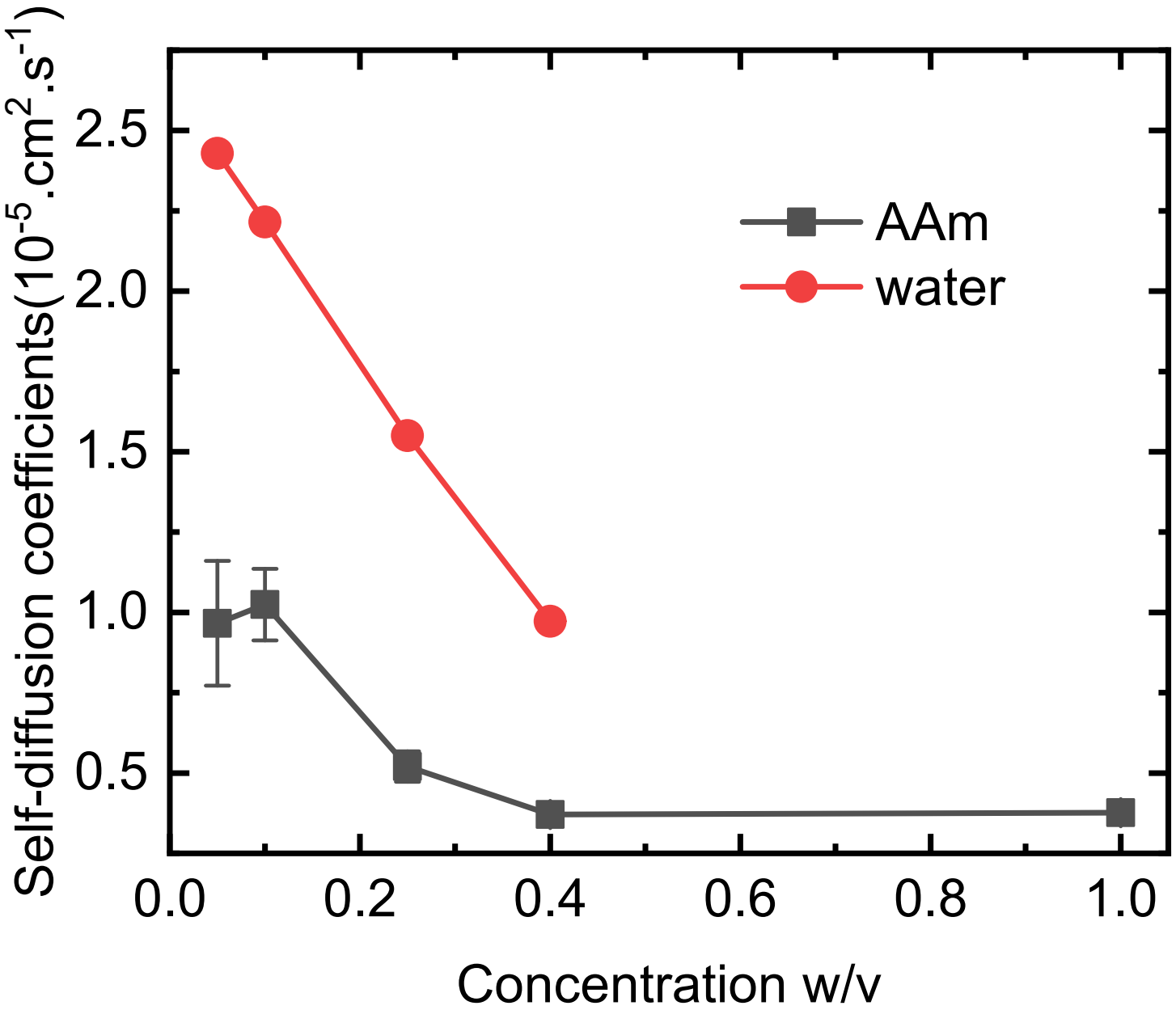

Figure S6. The self-diffusion coefficients in AAm aqueous solutions.

9. The fitted parameters of orientational autocorrlation functions for OW-HW bond

Table S2. The fitted parameters of orientational autocorrlation functions for OW-HW bond direction in AAm and PAAm aqueous solutions by tri-exponential decay functions.

OW-HW	w/v	A_1	τ_1 (ps)	A_2	τ_2 (ps)	A_3	τ_3 (ps)	$\tau_{average}$ (ps)
pure water		0.25	0.10	0.17	0.93	0.57	2.72	1.75
AAm	5%	0.26	0.11	0.24	1.22	0.50	3.21	1.91
AAm	10%	0.27	0.11	0.30	1.47	0.43	3.80	2.12
AAm	25%	0.28	0.12	0.37	2.13	0.35	6.24	2.99
AAm	40%	0.29	0.15	0.42	3.25	0.29	11.26	4.66
PAAm	5%	0.29	0.13	0.60	2.08	0.11	5.40	1.88

PAAm	10%	0.32	0.17	0.66	2.56	0.02	31.21	2.24
PAAm	25%	0.36	0.22	0.62	3.09	0.02	141.36	4.33
PAAm	40%	0.38	0.27	0.58	3.50	0.03	187.67	7.13

10. The fitted parameters of orientational autocorrlation functions for N-H bond

Table S3. The fitted parameters of orientational autocorrlation functions for N-H bond direction in AAm and PAAm aqueous solutions by tri-exponential decay functions.

OW-HW	w/v	A_1	τ_1 (ps)	A_2	τ_2 (ps)	A_3	τ_3 (ps)	$\tau_{average}$ (ps)
AAm	5%	0.18	0.14	0.17	2.96	0.65	8.70	6.18
AAm	10%	0.18	0.16	0.28	4.27	0.54	10.92	7.12
AAm	25%	0.20	0.20	0.38	7.09	0.42	18.92	10.68
AAm	40%	0.20	0.25	0.38	10.10	0.42	32.34	17.47
PAAm	5%	0.11	0.06	0.08	2.64	0.81	1035.82	839.23
PAAm	10%	0.17	0.19	0.10	11.54	0.73	1451.43	1060.73

PAAm	25%	0.12	7.75	0.10	234.94	0.78	8569.29	6708.47
PAAm	40%	0.11	12.46	0.07	306.47	0.82	13085.16	10752.65

11. The water-water mass-center radial distribution functions in AAm aqueous solutions.

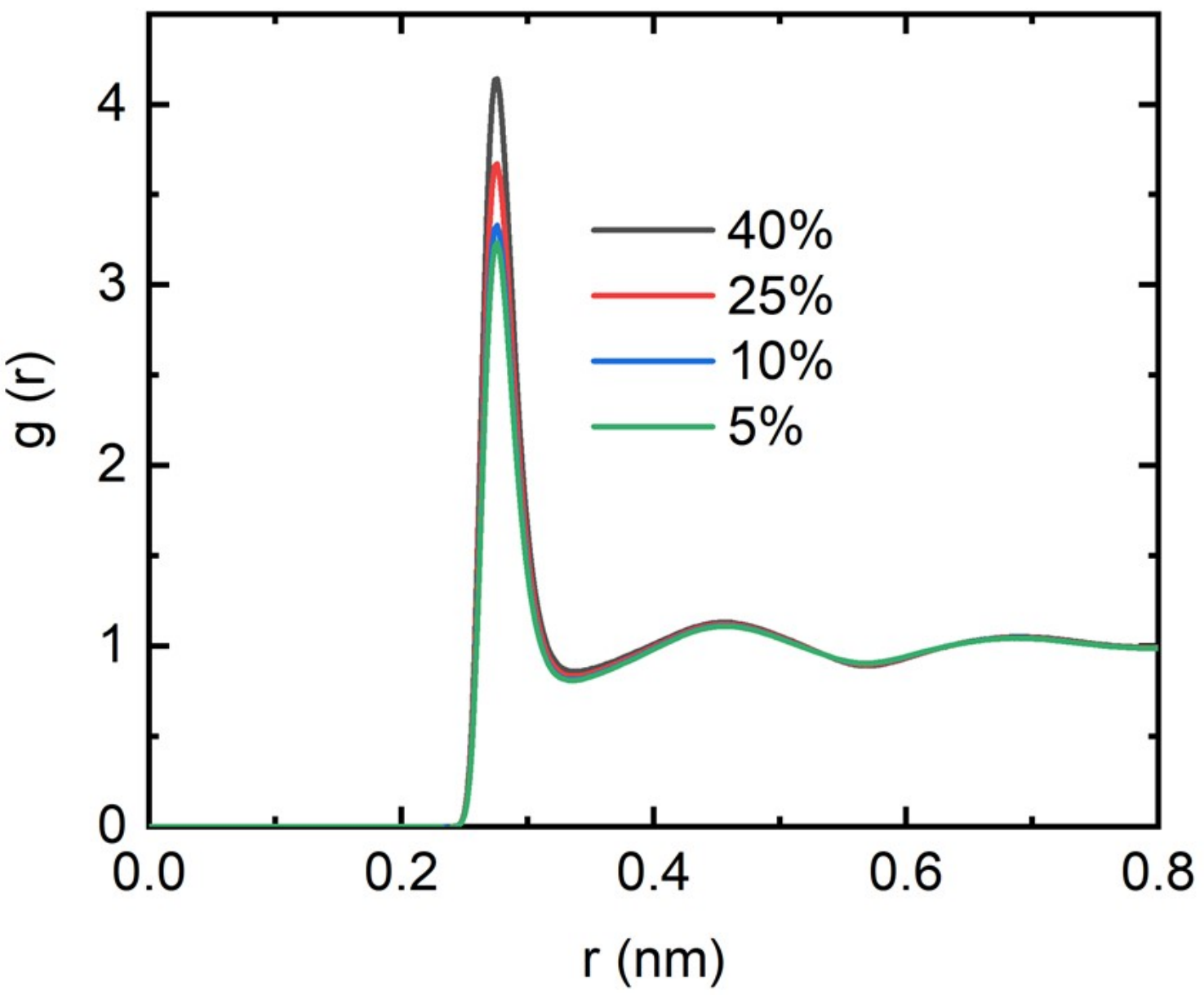

Figure S7. The water-water mass-center radial distribution functions.

12. The AAm-water mass-center radial distribution functions in AAm aqueous solutions.

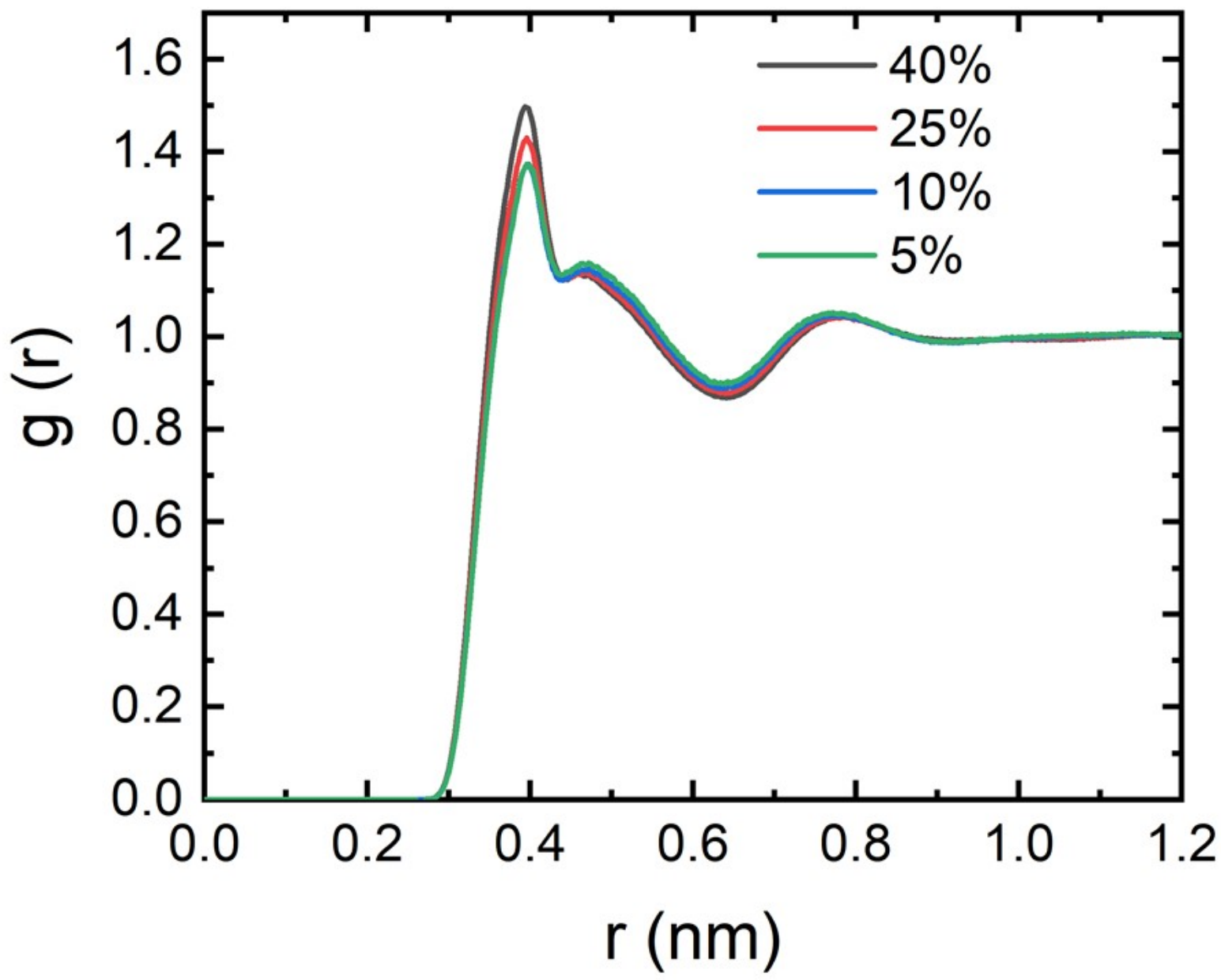

Figure S8. The AAm-water mass-center radial distribution functions.

13. The hydrodynamic model for AAm aqueous solutions.

To investigate whether the acrylamide/water mixture conforms to the hydrodynamic model, we used the Stokes-Einstein-Debye(SED) equation to fit the hydrodynamic model, substituting the slowest relaxation lifetime of the OKE TCF obtained from the simulation for the rotational lifetime. The SED equation given in eq S8.

$$\tau_{rot} = \frac{\eta V f_{\theta} I_c}{k_B T} \qquad \text{(S8)}$$

where τ_{rot} is single-molecule rotational lifetime, η is the shear viscosity, V is the solvent excluded volume 58Å^3, f_θ is 1.15, I_c is an interaction, usually the stick boundary conditions asks I_c=1. The shear viscosity is from Stephen Van Wyck and Michael Fayer.[*J. Phys. Chem. B*, 2023, *127*, 1276-1286.] As shown in Figure S9, we can clearly see that the OKE

relaxation lifetime obtained from the simulation is very close to the rotational lifetime derived from the SED model and follows a typical linear trend. This indicates that, within the concentration range studied, the AAm/water mixture is well described by the hydrodynamic model.

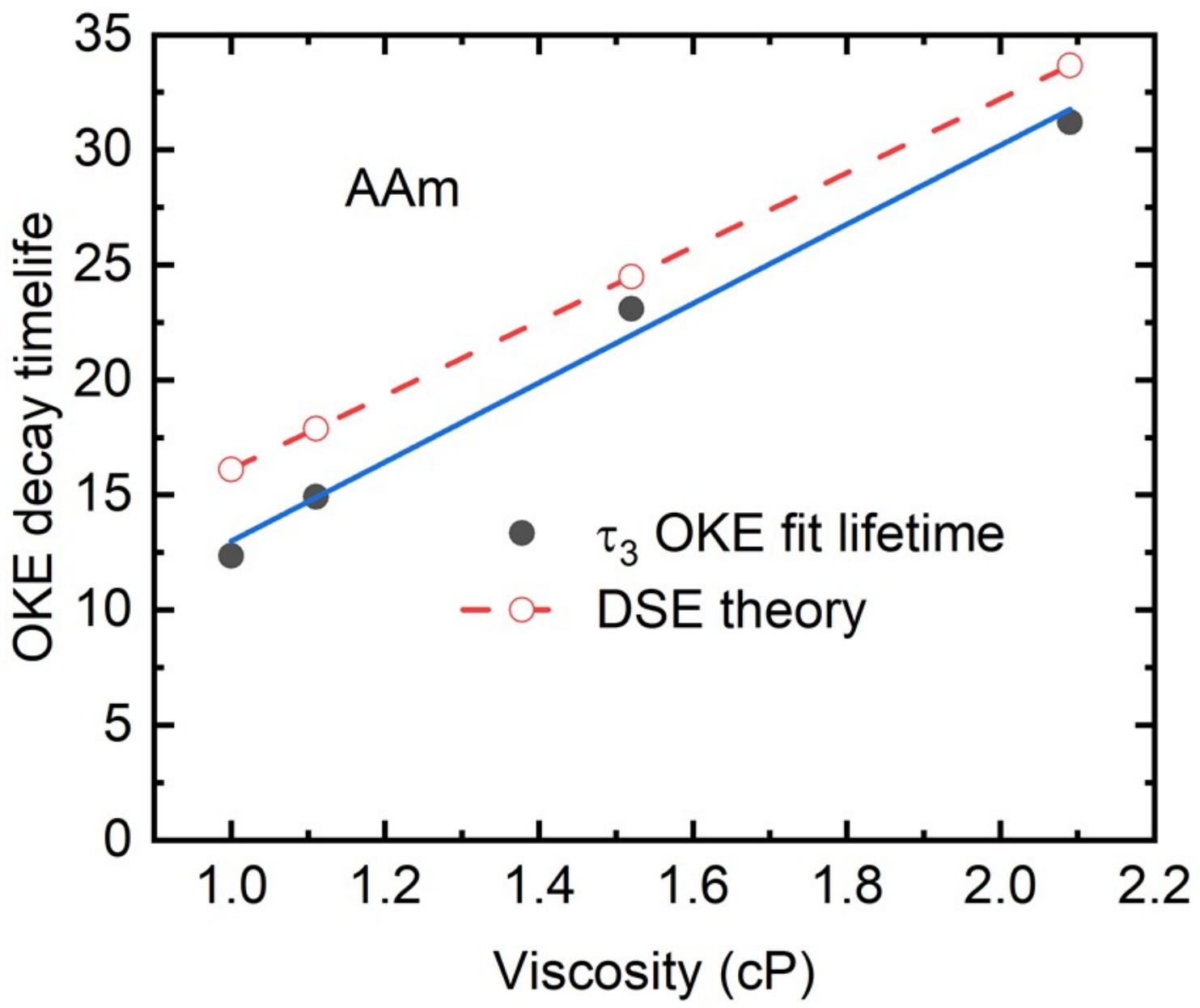

Figure S9. The hydrodynamic model for AAm aqueous solutions.

14. The comparison of OKE spectrum between the simulation and experiment in PAAm aqueous solutions.

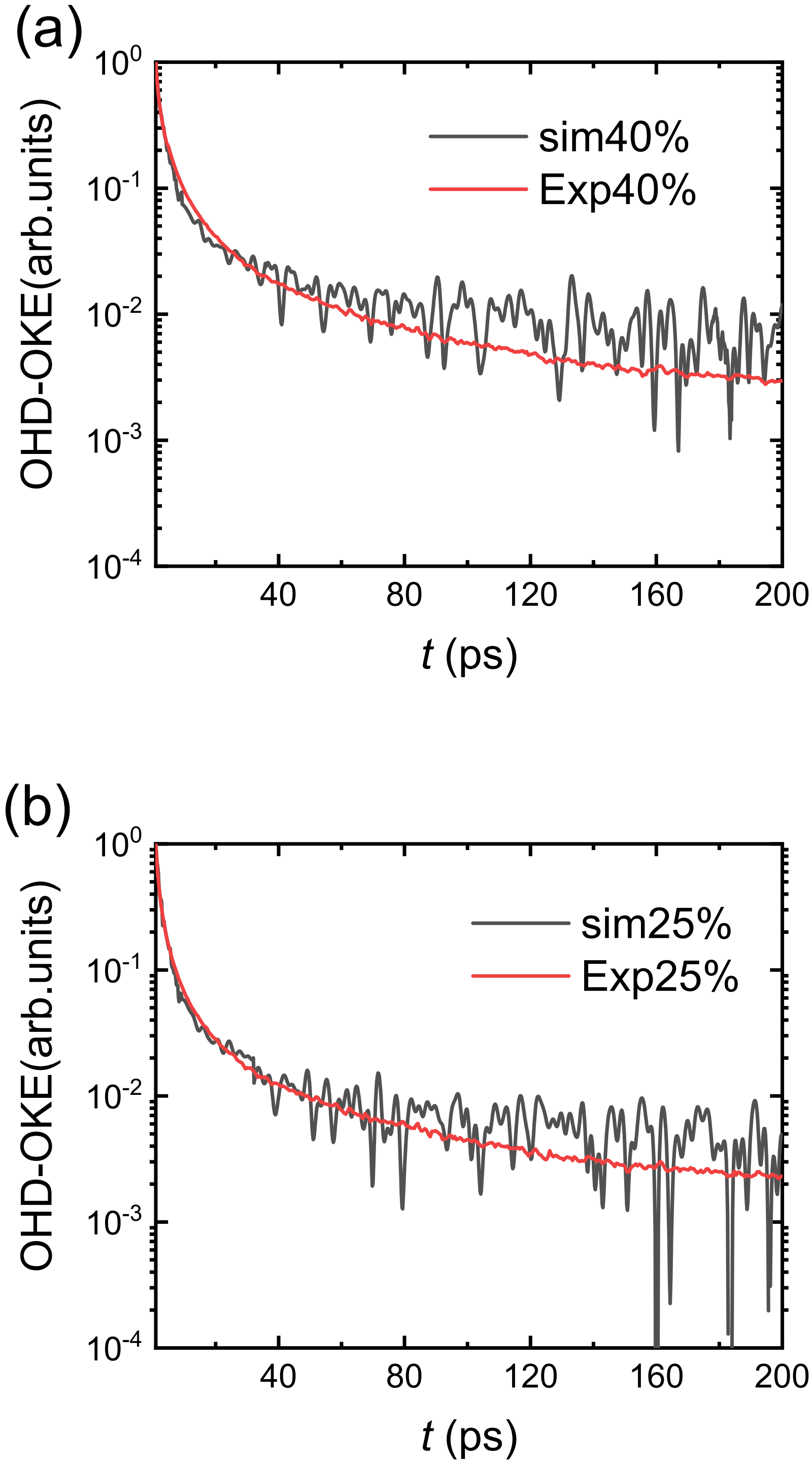
(a)
sim40%
Exp40%
OHD-OKE(arb.units)
10^0
10^{-1}
10^{-2}
10^{-3}
10^{-4}
40
80
120
160
200
t (ps)
(b)
sim25%
Exp25%
OHD-OKE(arb.units)
10^0
10^{-1}
10^{-2}
10^{-3}
10^{-4}
40
80
120
160
200
t (ps)

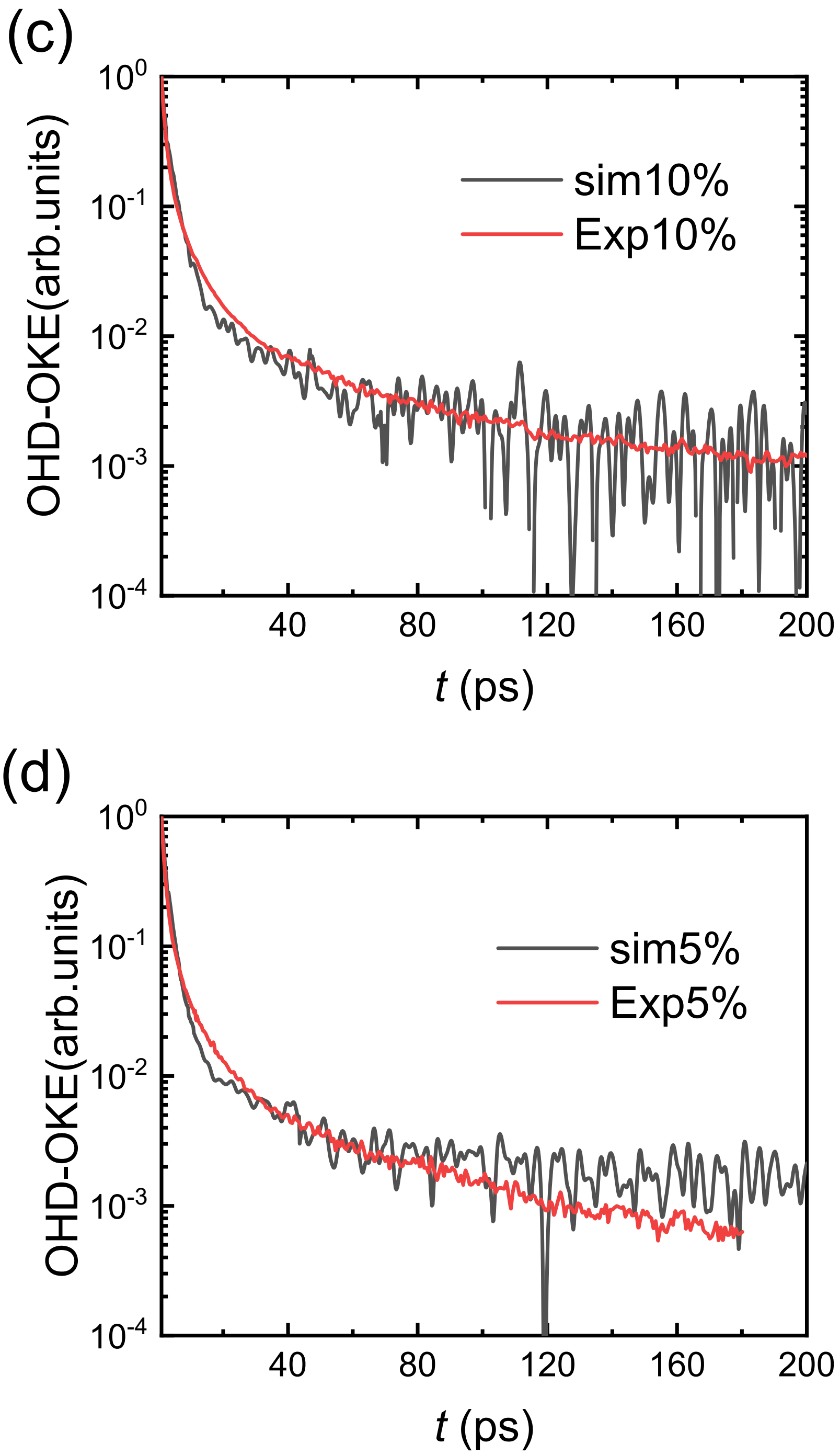

Figure S10. The comparison of OKE spectrum between the simulation and experiment in PAAm aqueous solutions. (a) 40% v/w. (b) 25% v/w. (c) 10% v/w. (d) 5% v/w.

15. The diffusion coefficients of water and PAAm in PAAm aqueous solutions.

Table S4. The diffusion coefficients of water and PAAm in PAAm aqueous solutions.

	Pure water	5%	10%	25%	40%
D_{water}(10^{-5} $cm^2.s^{-1}$)	2.5238	2.5215	2.2933	1.8552	1.5932
D_{PAAM}(10^{-5} $cm^2.s^{-1}$)		0.1188	0.0740	0.0778	0.0053

16. The orientational ACFs for N-H bond in PAAm.

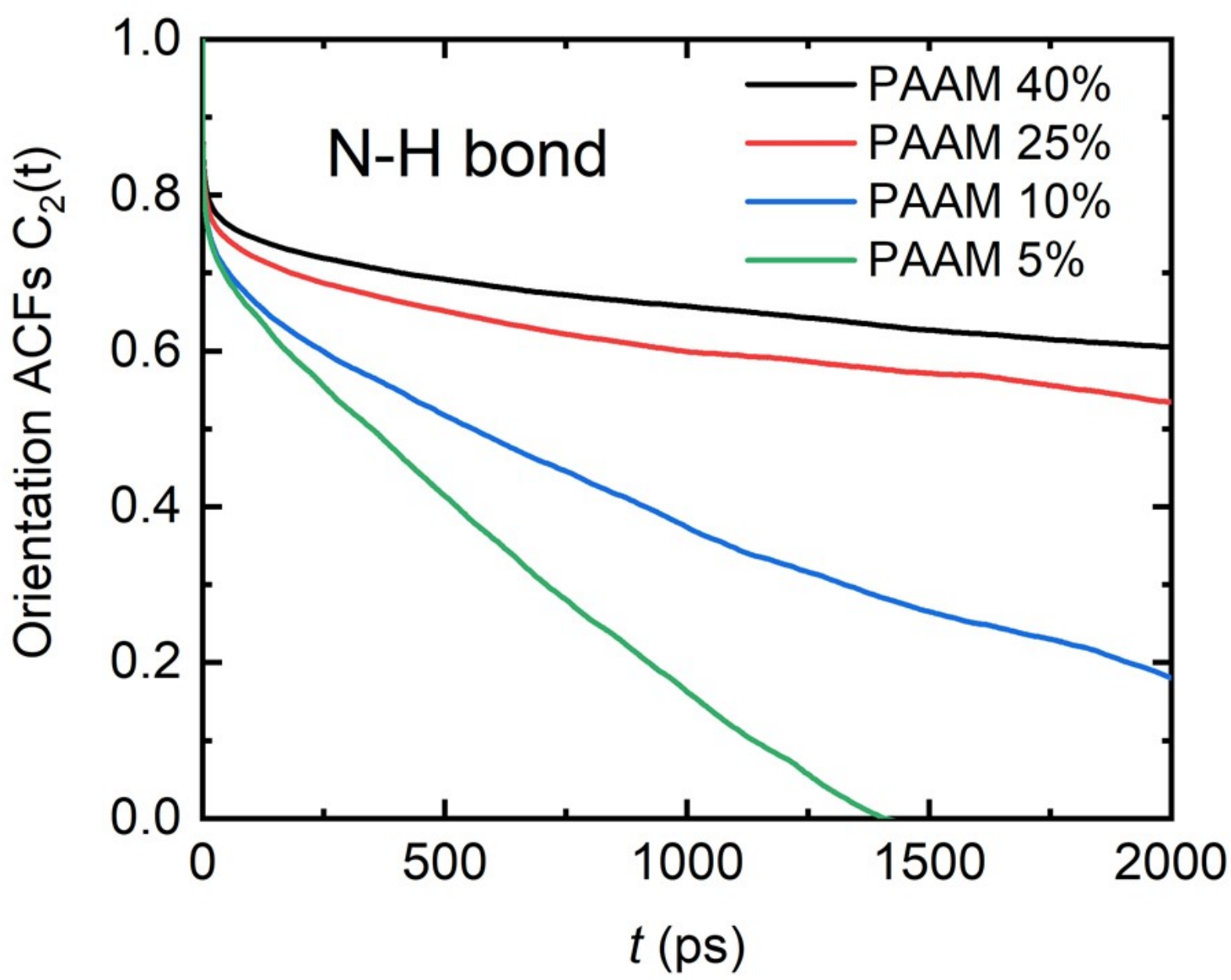

Figure S11. The orientational ACFs for N-H bond in PAAm.

17. The orientational ACFs of PAAm chain.

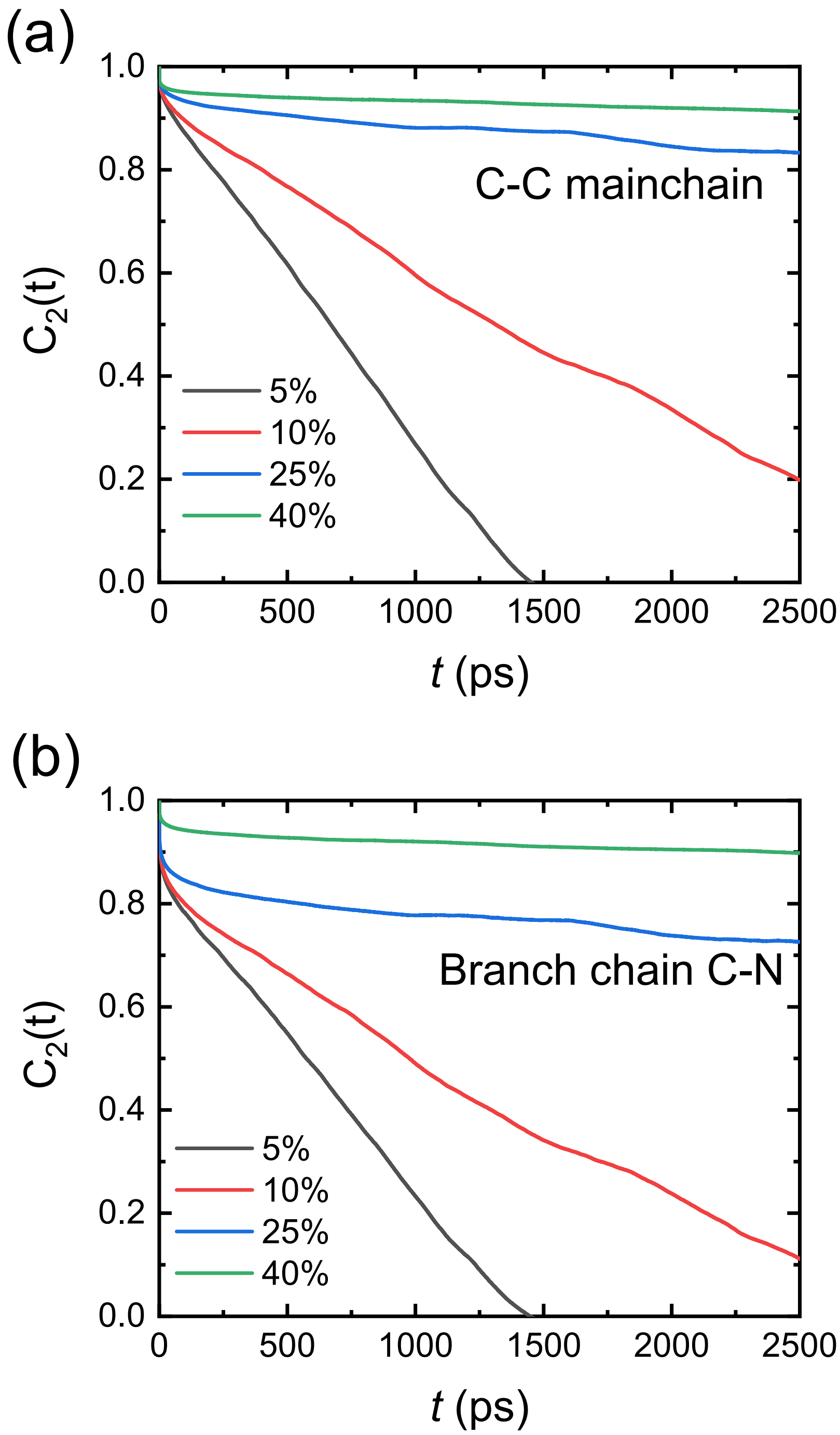

Figure S12 The orientational ACFs of PAAm chain. (a) C-C main chain. (b) C-N branch chain.

18. The relationship between the orientational ACFs of OW-HW bond and the diffusion coefficients of water.

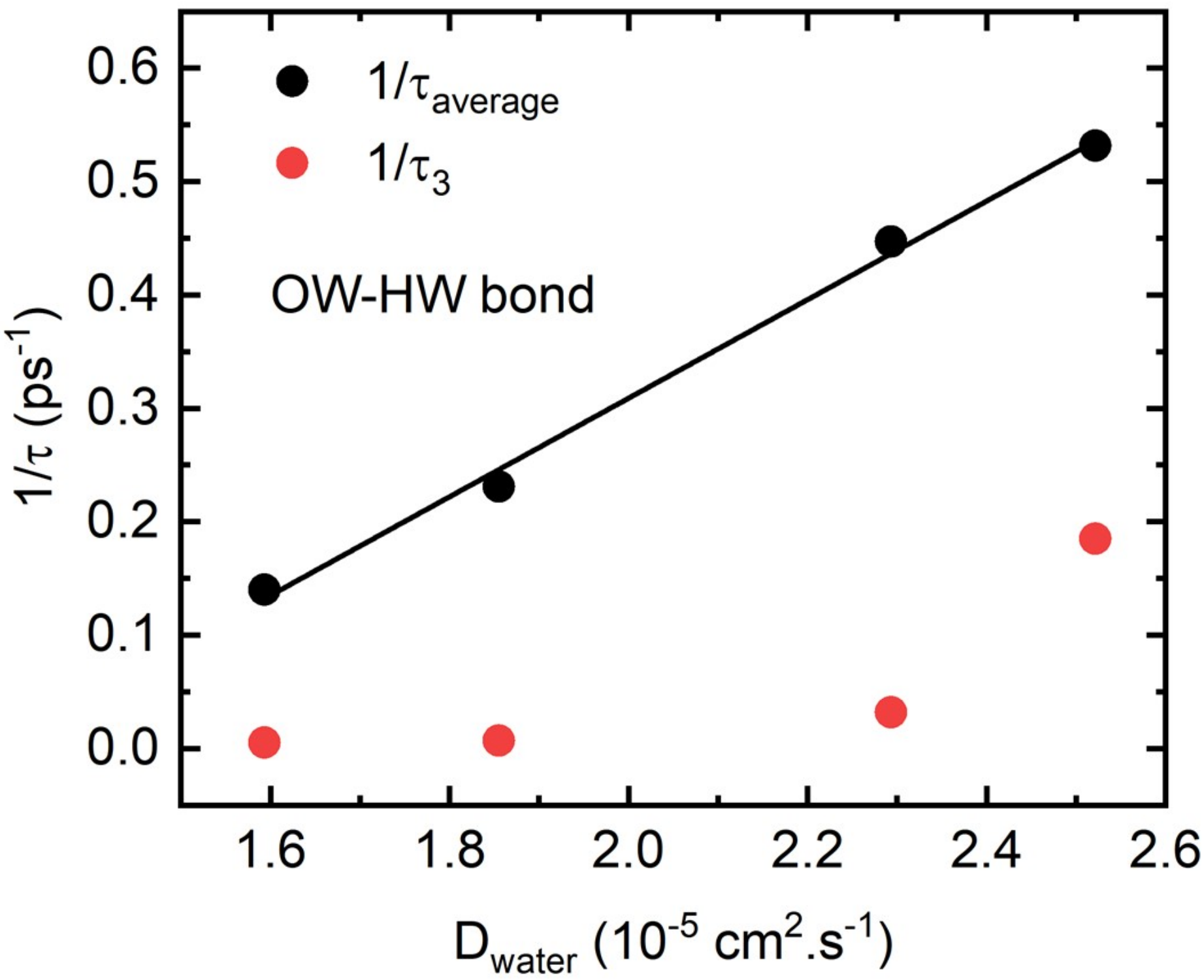

Figure S13. The relationship between the fitted relaxation lifetime for orientational ACFs of OW-HW bond and the diffusion coefficients of water.

19. The hydrogen bonding ACFs of N-H(PAAm)...OW(water)

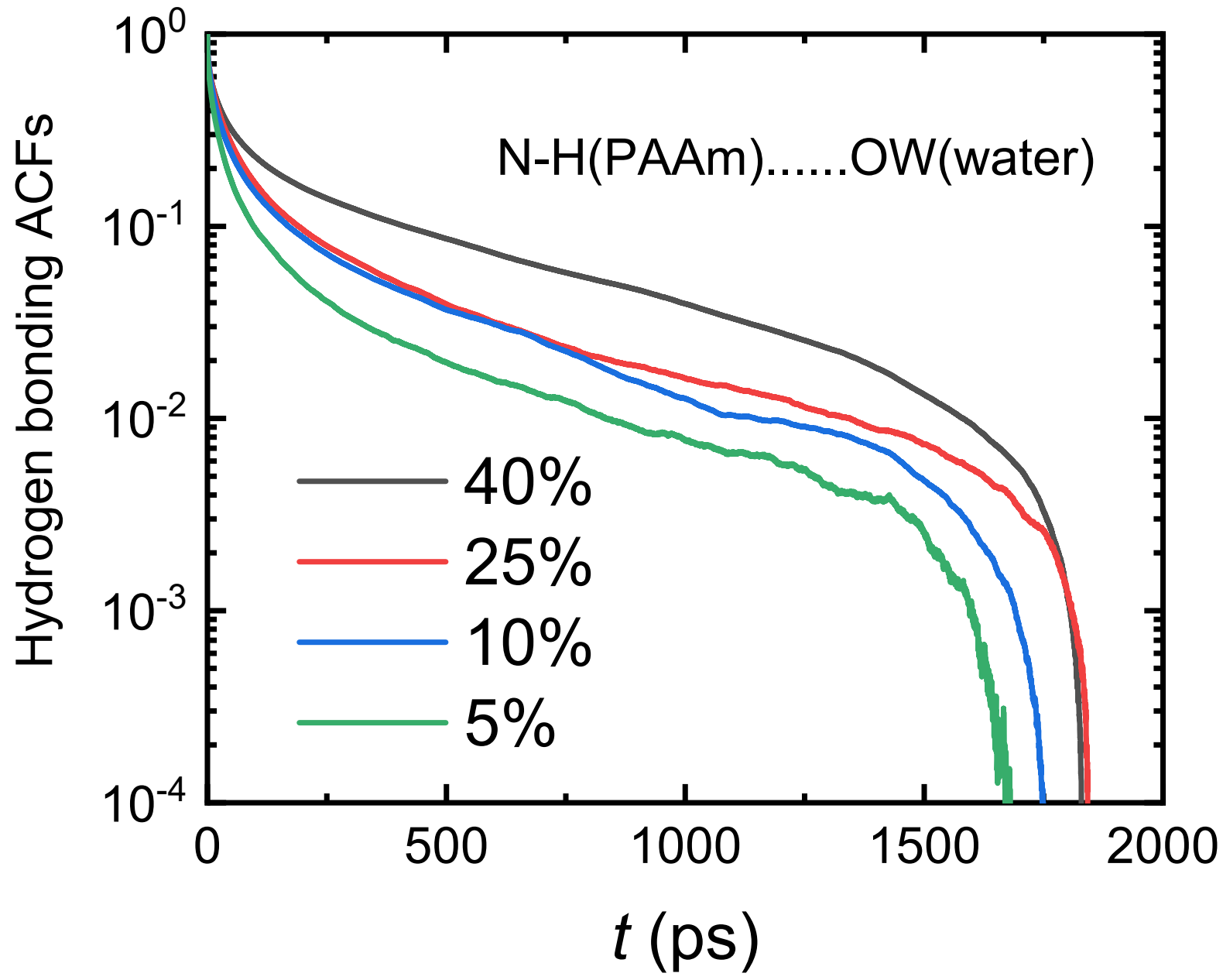

Figure S14. The hydrogen bonding ACFs of N-H(PAAm)...OW(water) in PAAm aqueous solutions.

20. The hydrogen bonding ACFs of OW-HW(water)...O(PAAm)

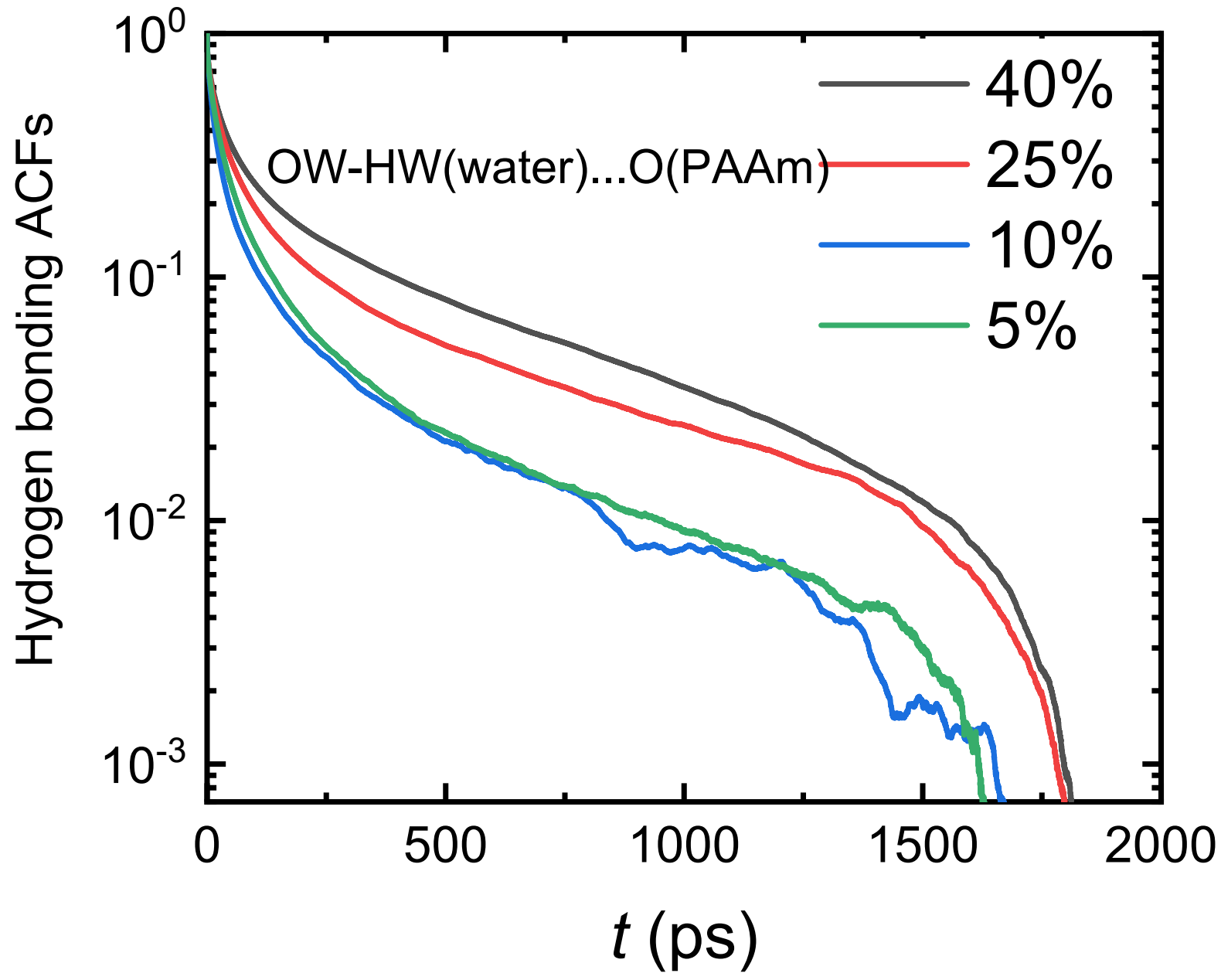

Figure S15. The hydrogen bonding ACFs of OW-HW(water)...O(PAAm) in PAAm aqueous solutions.

21. The concentration dependent solvation area of PAAm.

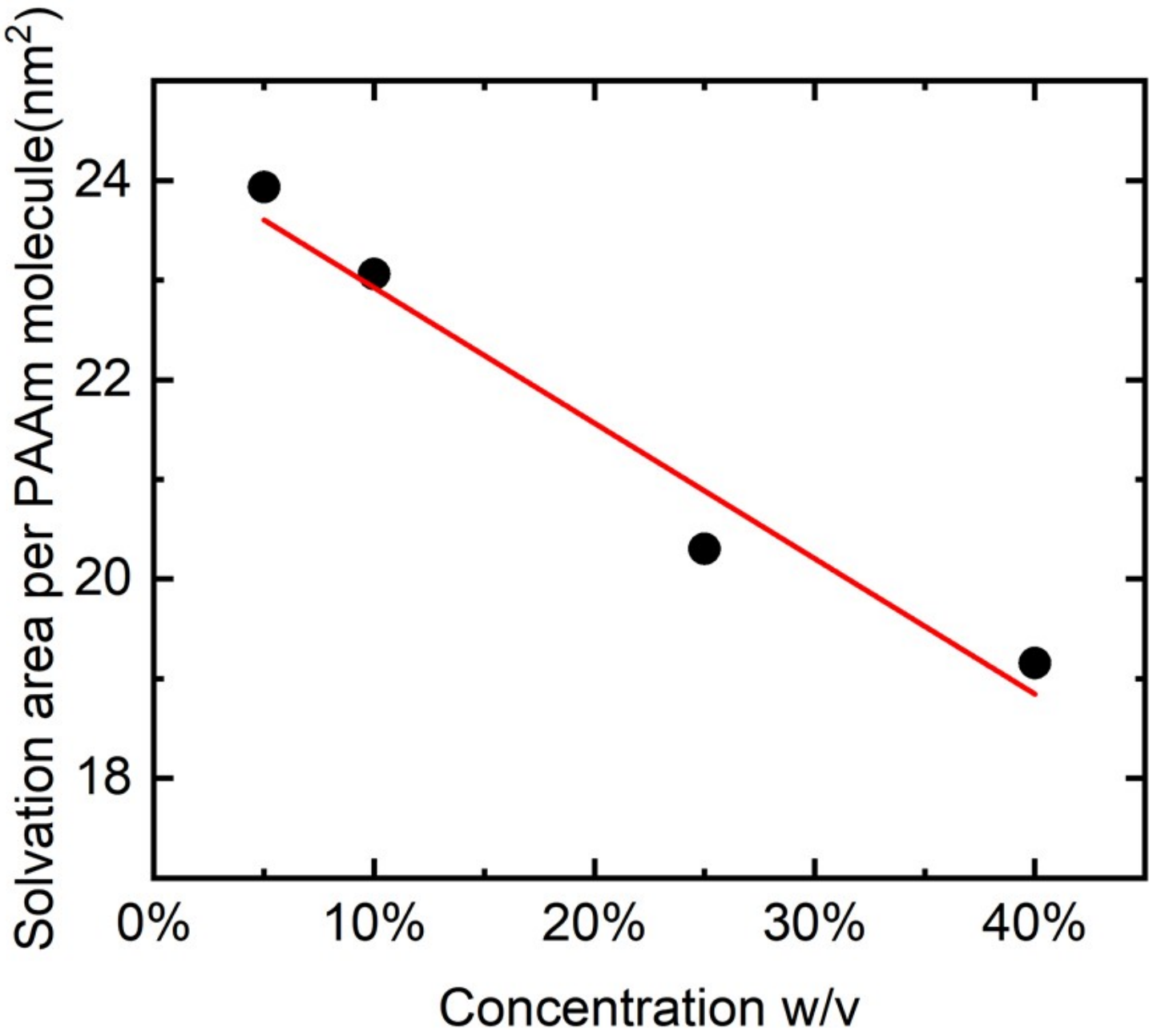

Figure S16. The concentration dependent solvation area per PAAm molecule.
